# Minimal CBX3-Derived UCOE Confers Long-Term Resistance to Transgene Silencing in Human iPSCs During Neuronal Differentiation

**DOI:** 10.3390/ijms27188046

**Published:** 2026-09-10

**Authors:** Omer Faruk Anakok, Guven Akcay

**Affiliations:** 1 Department of Medical Biology, Faculty of Medicine, Bolu Abant İzzet Baysal University, Bolu 14030, Turkey; 2 Department of Biophysics, Faculty of Medicine, Bolu Abant İzzet Baysal University, Bolu 14030, Turkey; guvenakcayibu@gmail.com

**Keywords:** universal chromatin opening element (UCOE), gene silencing, human pluripotent stem cells (iPSCs), lentiviral transduction, epigenetic stability

## Abstract

Long-term transgene silencing remains a major challenge in lentiviral gene delivery, particularly in pluripotent stem cells undergoing lineage-specific differentiation. Universal chromatin opening elements (UCOEs) have emerged as effective regulatory elements for protecting transgene expression against epigenetic silencing; however, their relatively large-size limits vector design flexibility. In the present study, we evaluated the anti-silencing activity of three next-generation UCOE constructs (1.7 kb, 1.2 kb, and a newly developed minimal 0.5 kb fragment) during long-term culture and neuronal differentiation of human induced pluripotent stem cells (iPSCs). UCOE fragments were cloned into self-inactivating lentiviral vectors and validated by restriction enzyme analysis and agarose gel electrophoresis. Lentiviral particles produced in HEK293T cells were used to transduce human iPSCs, followed by neuronal differentiation. Transgene expression was monitored for up to 60 days using fluorescence microscopy, confocal immunofluorescence, flow cytometry, and vector copy number analysis by quantitative PCR. All UCOE-containing vectors demonstrated significantly improved transgene stability compared with the UCOE-less control throughout both the undifferentiated and differentiated stages. The UCOE-containing constructs maintained substantially greater expression stability than the UCOE-less control, with the minimal 0.5 kb UCOE showing long-term performance comparable to the larger UCOE constructs. These findings demonstrate that considerable UCOE minimization can be achieved without markedly compromising anti-silencing activity, providing a potential vector-design advantage by reducing the regulatory cassette footprint and increasing available vector capacity. The compact 0.5 kb UCOE therefore represents a promising regulatory element for sustained transgene expression in stem cell engineering, disease modeling, regenerative medicine, and future gene therapy applications. However, the present study did not directly assess genomic safety, insertional effects, genotoxicity, or cellular transformation, and dedicated preclinical studies will be required to determine the long-term safety and in vivo performance of this compact UCOE configuration.

## 1. Introduction

Gene therapy has emerged as one of the most promising strategies for the treatment of inherited, acquired, and degenerative diseases by enabling the delivery of therapeutic nucleic acids into target cells. Viral and non-viral delivery systems have been extensively developed to achieve efficient and long-term transgene expression. Among these approaches, viral vectors remain the most effective because of their high gene transfer efficiency, whereas non-viral systems generally offer improved safety but lower transfection efficiency. Regardless of the delivery platform employed, sustained and predictable transgene expression remains one of the principal challenges limiting the long-term efficacy of gene therapy applications [1,2,3]. Lentiviral vectors are widely used in gene therapy because they efficiently transduce both dividing and non-dividing cells while stably integrating the therapeutic cassette into the host genome. These characteristics make them particularly attractive for applications involving long-lived somatic cells, neural tissues, hematopoietic stem cells, and pluripotent stem cells. In addition, their relatively large packaging capacity and compatibility with tissue-specific regulatory elements have further expanded their use in both experimental and clinical settings [2,3,4,5]. Despite these advantages, integrated transgenes frequently undergo progressive transcriptional silencing during prolonged culture or cellular differentiation, primarily due to DNA methylation, repressive histone modifications, chromatin condensation, and position-effect variegation [3,4,5,6]. Such epigenetic silencing remains a major obstacle for durable therapeutic gene expression and limits the clinical potential of lentiviral vector-based gene therapy.

To overcome transcriptional silencing, ubiquitous chromatin opening elements (UCOEs) have been developed as regulatory DNA elements capable of maintaining an open chromatin configuration around integrated transgene. UCOEs originate from CpG island-associated housekeeping gene loci and exhibit remarkable resistance to DNA methylation and repressive chromatin remodeling. Among the currently available UCOEs, the HNRPA2B1-CBX3-derived A2UCOE has become the most extensively characterized element and has consistently demonstrated the ability to support long-term transgene expression across multiple cell types, including pluripotent stem cells, differentiated somatic cells, and several in vivo disease models [6,7,8,9,10,11,12,13,14,15,16,17,18]. In addition to preserving stable expression, A2UCOE-containing vectors have shown improved biosafety by functioning independently of strong viral enhancer elements, thereby reducing the potential risk of insertional mutagenesis associated with conventional promoter–enhancer combinations [7,11,12,17]. The therapeutic value of UCOEs has been demonstrated in numerous experimental and translational studies involving inherited and acquired diseases. Stable transgene expression mediated by A2UCOE-containing vectors has been reported in gene therapy approaches for chronic granulomatous disease, pulmonary alveolar proteinosis, primary immunodeficiencies, cystic fibrosis, sickle cell disease, thalassemia, and regulatory T-cell engineering. Furthermore, these elements have become valuable tools for recombinant protein production because they reduce expression variability during long-term cell culture. Consequently, optimization of UCOE architecture has become an active area of investigation aimed at improving both therapeutic efficacy and vector safety [19,20,21,22,23,24,25,26].

Several groups have attempted to identify smaller functional UCOE fragments while preserving their anti-silencing properties. Progressive truncation studies demonstrated that sub-fragments derived from the HNRPA2B1-CBX3 locus could retain chromatin-opening activity despite substantial reductions in size. Fragments ranging from approximately 1.2 to 1.7 kb have been reported to maintain stable expression in pluripotent and multipotent stem cells, whereas additional studies suggested that CpG-rich regions located within the CBX3 locus may contribute significantly to UCOE function [11,17,24,27]. These observations indicate that the complete 2.2 kb A2UCOE sequence may not be essential for sustained transgene expression and that compact regulatory elements could provide important advantages by increasing vector packaging capacity and simplifying vector design. Nevertheless, several important questions remain unresolved. Although multiple truncated A2UCOE derivatives have been described, it is still unclear whether a minimal promoterless CpG-rich fragment derived from the CBX3 locus alone is sufficient to maintain long-term transgene expression in human induced pluripotent stem cells during lineage-specific differentiation. Moreover, the functional performance of such compact UCOEs has not been comprehensively evaluated throughout neuronal differentiation, a process during which epigenetic remodeling frequently induces transgene silencing [14,17,24,27,28]. Addressing this knowledge gap is particularly important for gene therapy strategies targeting the nervous system, where persistent and stable transgene expression is essential for long-term therapeutic efficacy.

In the present study, we evaluated two optimized A2UCOE derivatives (1.7 kb and 1.2 kb) together with a novel 0.5 kb CpG-rich fragment derived from the CBX3 locus using lentiviral vectors in human induced pluripotent stem cells. Transgene stability was monitored in both undifferentiated iPSCs and throughout neuronal differentiation by assessing eGFP expression and vector copy number over an extended culture period. We hypothesized that this compact promoterless CBX3-derived fragment would retain anti-silencing activity comparable to or greater than larger UCOE constructs while providing a smaller and potentially safer regulatory element for future gene therapy and recombinant protein production applications.

## 2. Results

### 2.1. Construction and Molecular Validation of Lentiviral UCOE Constructs

To investigate the ability of different ubiquitous chromatin opening element (UCOE) fragments to maintain stable transgene expression, three lentiviral vectors carrying distinct UCOE sequences were successfully generated using a common self-inactivating lentiviral backbone. The constructs comprised a 1.7 kb UCOE spanning the HNRPA2B1–CBX3 dual-promoter region, a 1.2 kb truncated derivative of the same region, and a newly designed minimal 0.5 kb CBX3-derived UCOE. Two reference vectors were included throughout the study: a UCOE-less vector (negative control) and the previously characterised full-length A2UCOE-SEW vector (positive control).

**Figure 1:**
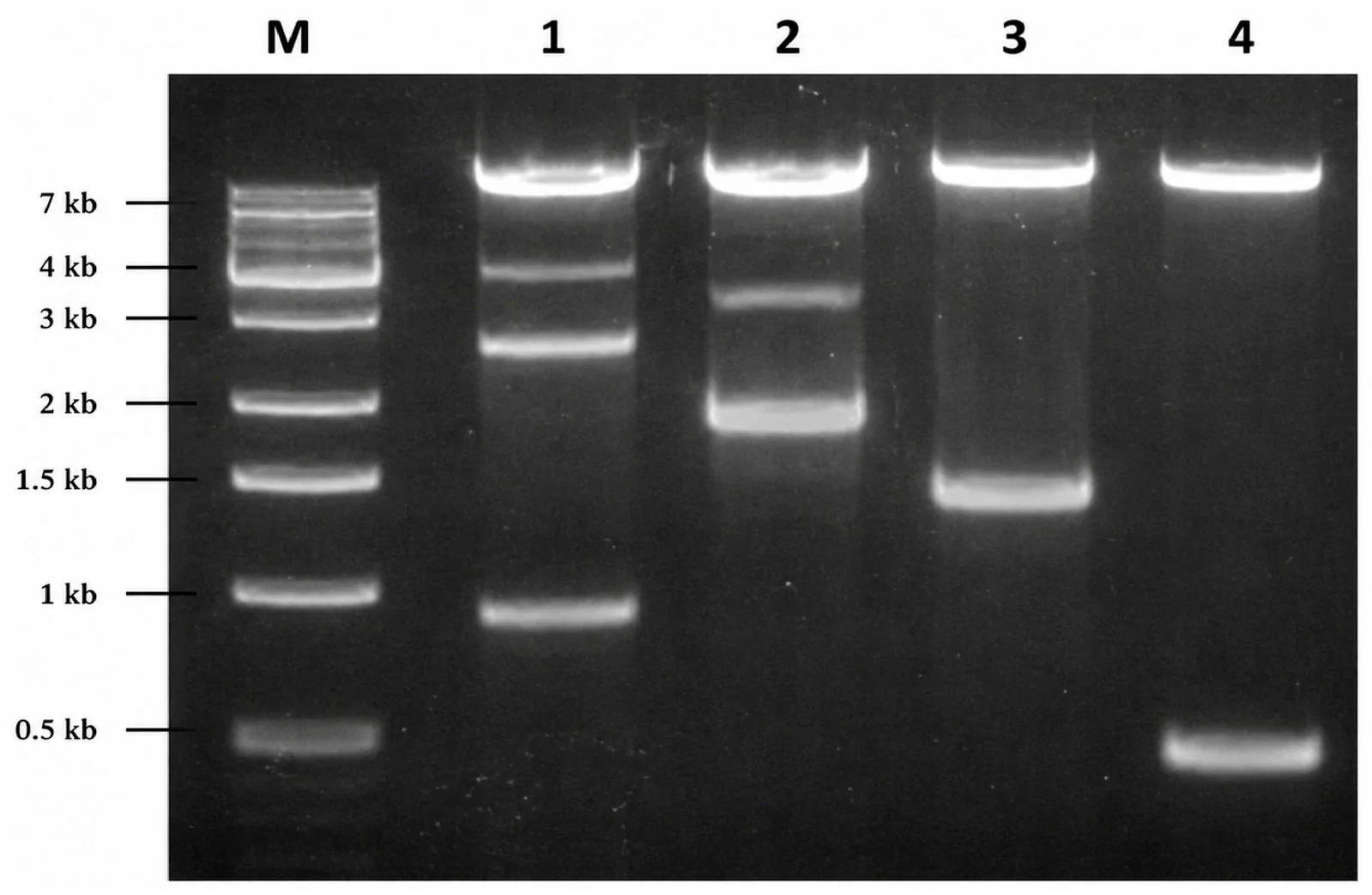
Molecular validation of the lentiviral UCOE constructs by restriction enzyme analysis. Representative agarose gel electrophoresis of analytical restriction digests of the lentiviral transfer plasmids carrying the 1.7 kb, 1.2 kb, or 0.5 kb UCOE fragments, together with the UCOE-less control. Digestion with SfiI released the inserted UCOE fragment from the common vector backbone, and complementary digests with EcoRV, ClaI, BamHI, and AgeI were used to confirm insert identity, orientation, and structural integrity. Fragment sizes were estimated against 100 bp and 1 kb DNA ladders (New England Biolabs, Ipswich, MA, USA). In all constructs the observed banding patterns matched the predicted restriction maps, with no evidence of rearrangement or non-specific recombination.

Successful cloning of the UCOE fragments was assessed by restriction enzyme digestion followed by agarose gel electrophoresis. Digestion with SfiI generated fragments corresponding to the expected insert sizes and vector backbone, while complementary digestions with EcoRV, ClaI, BamHI, and AgeI produced banding patterns consistent with the predicted restriction maps (Figure 1). These analyses supported the expected size, orientation, and overall structural organization of the cloned fragments. To confirm the nucleotide-level integrity of the newly developed 0.5 kb UCOE construct, the final recombinant lentiviral transfer plasmid was subjected to Sanger sequencing. Five overlapping amplicons covering the 5′ vector–0.5UCOE junction (432 bp), the internal 0.5 kb UCOE region (512 bp forward and 509 bp reverse), the 0.5UCOE–PGK promoter junction (478 bp), the PGK–eGFP junction (556 bp), and the eGFP–WPRE junction (501 bp) were analyzed. All sequenced regions showed 100% identity with the corresponding reference design, with no nucleotide substitutions, insertions, or deletions detected. Representative sequence alignment summaries and electropherogram traces are presented in Supplementary Figure S1.

**Figure 2:**
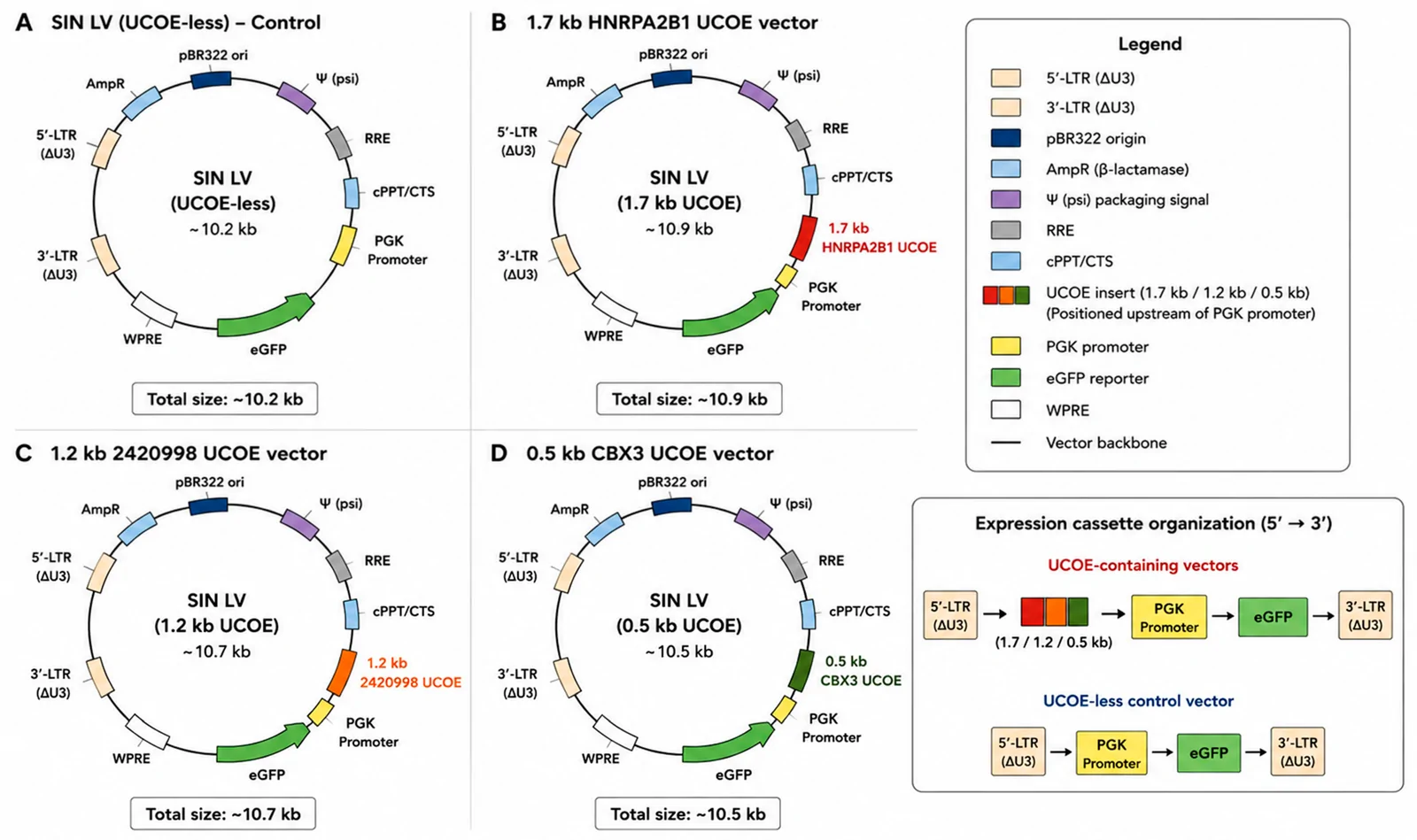
Schematic representation and structural maps of lentiviral vectors carrying different UCOEs. All constructs are self-inactivating (SIN) lentiviral vectors containing an identical backbone comprising the pBR322 origin of replication, ampicillin resistance gene (AmpR), internal PGK promoter, and eGFP reporter cassette. (**A**) The UCOE-less control vector lacking any UCOE element (~10.2 kb). In the UCOE-containing vectors (**B**–**D**), a specific UCOE (1.7 kb HNRPA2B1, 1.2 kb 2420998, or 0.5 kb CBX3) is positioned upstream of the PGK promoter. This strategic placement drives long-term, stable transgene expression by protecting the promoter and reporter gene from epigenetic silencing, enabling a direct comparison of how UCOE size influences vector performance while maintaining identical architecture.

Schematic plasmid maps illustrating the structural organization of all lentiviral constructs are presented in Figure 2. All vectors shared an identical backbone containing the bacterial origin of replication, ampicillin resistance cassette, phosphoglycerate kinase (PGK) promoter, enhanced green fluorescent protein (eGFP) reporter cassette, and self-inactivating lentiviral long terminal repeats. In the UCOE-containing constructs, the respective UCOE fragments were positioned immediately downstream of the PGK promoter and upstream of the eGFP coding sequence, resulting in a linear expression cassette organized as PGK promoter → UCOE → eGFP. The UCOE-less control vector retained the corresponding PGK–eGFP expression cassette without an intervening UCOE. The only structural difference among the constructs was therefore the incorporation of the respective UCOE fragment between the PGK promoter and the eGFP reporter gene, enabling direct comparison of their effects on long-term transgene expression while minimizing vector-related variability.

### 2.2. Characterization of Human iPSCs and Evaluation of Lentiviral Transduction

Before investigating the long-term effects of UCOEs on transgene expression, the morphology, reporter expression, and pluripotency-associated phenotype of human induced pluripotent stem cells (iPSCs) were evaluated following lentiviral transduction. Untransduced human iPSCs were included as a negative control and were maintained and analyzed in parallel with lentivirally transduced cultures under the same experimental conditions.

**Figure 3:**
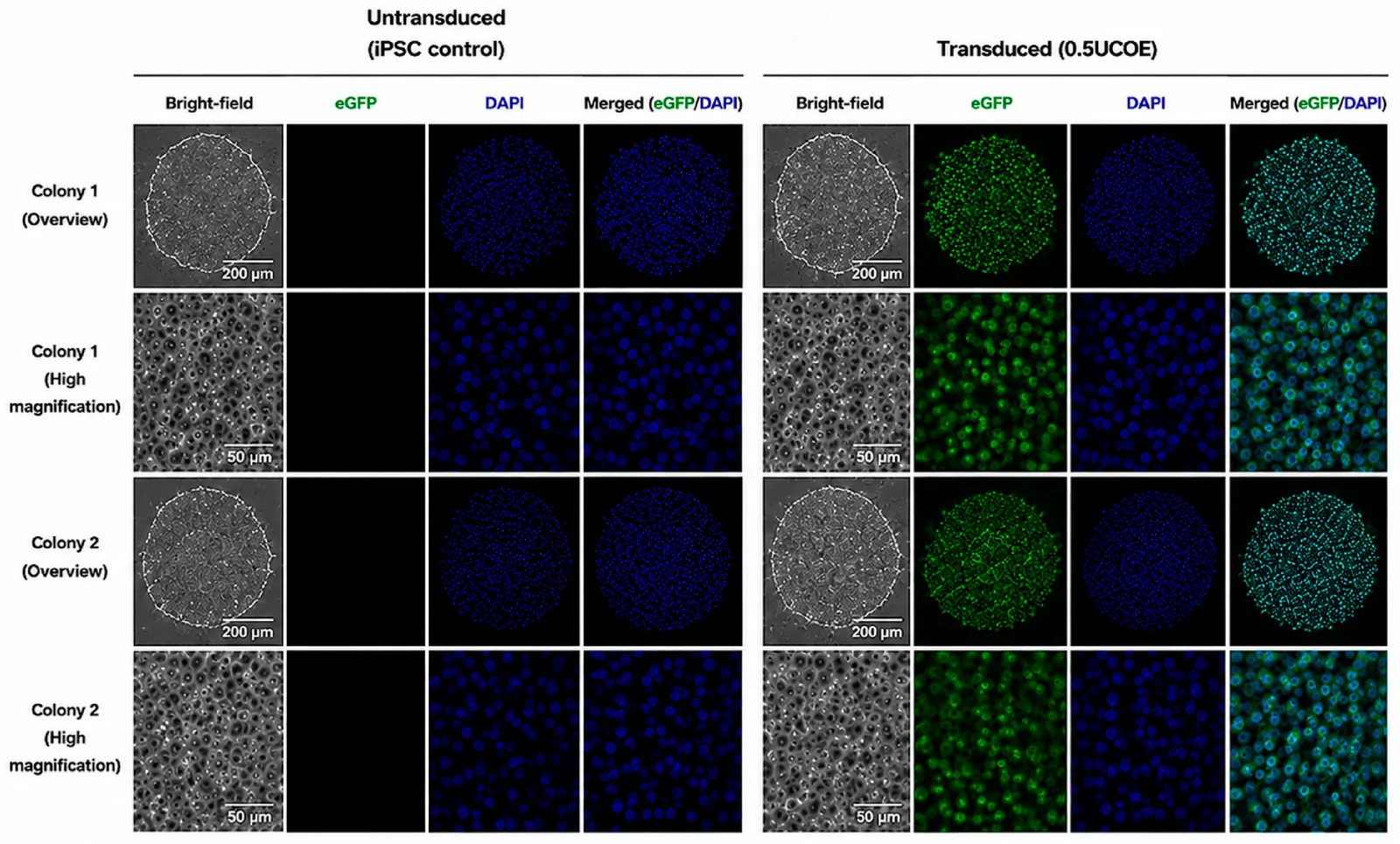
Morphological and fluorescence characterization of lentivirally transduced human induced pluripotent stem cells. Representative bright-field, eGFP fluorescence, DAPI nuclear staining, and merged eGFP/DAPI images of untransduced control and lentivirally transduced undifferentiated human iPSC colonies. Overview images demonstrate the characteristic compact colony morphology, with the untransduced control showing no eGFP signal and transduced colonies exhibiting robust eGFP reporter expression throughout. Higher-magnification images show the typical high nucleus-to-cytoplasm ratio and closely packed cellular organization of undifferentiated iPSCs, together with homogeneous reporter expression in the transduced group. DAPI staining demonstrates the distribution and morphology of nuclei, while merged images illustrate eGFP expression within the densely organized colonies. Scale bars = 200 µm for overview images and 50 µm for high-magnification images.

Bright-field microscopy demonstrated that both untransduced and lentivirally transduced iPSCs retained the characteristic morphology of undifferentiated pluripotent stem cells, forming compact colonies with well-defined borders and a high nucleus-to-cytoplasm ratio. Untransduced iPSCs showed no detectable eGFP fluorescence, whereas transduced cultures displayed robust eGFP fluorescence throughout the colonies. DAPI staining demonstrated comparable nuclear organization in both groups. Confocal microscopy further demonstrated a homogeneous distribution of eGFP fluorescence within transduced colonies while preserving normal colony architecture. No obvious alterations in colony morphology, excessive cell detachment, or structural abnormalities were observed following lentiviral transduction (Figure 3).

**Figure 4:**
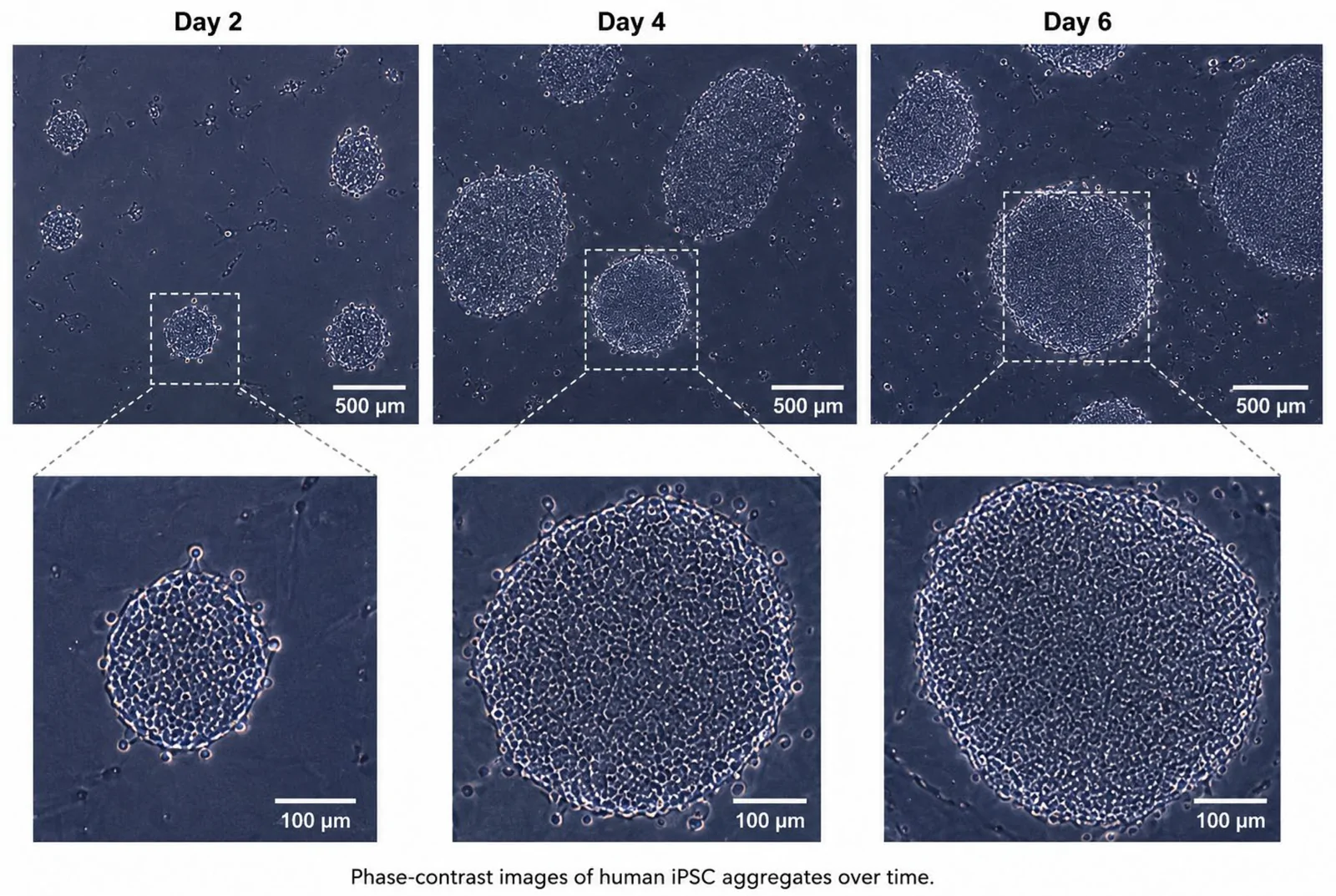
Expansion of transduced human iPSC colonies during the pre-differentiation culture period. Representative phase-contrast microscopy images of transduced human iPSC colonies at Days 2, 4, and 6 of culture. Upper panels show low-magnification overview fields demonstrating progressive increases in colony size and density over time. Dashed boxes indicate the regions displayed at higher magnification in the corresponding lower panels. Higher-magnification images demonstrate preservation of compact colony organization, well-defined colony borders, and the characteristic high nucleus-to-cytoplasm ratio of undifferentiated human iPSCs throughout the expansion period. Scale bars = 500 µm for overview images and 100 µm for high-magnification images.

The developmental progression of cultured iPSCs was also monitored during the initial expansion period prior to differentiation. Phase-contrast microscopy demonstrated progressive colony enlargement from Days 2 to 6 while maintaining the characteristic compact morphology and colony organization of undifferentiated pluripotent stem cells (Figure 4).

**Figure 5:**
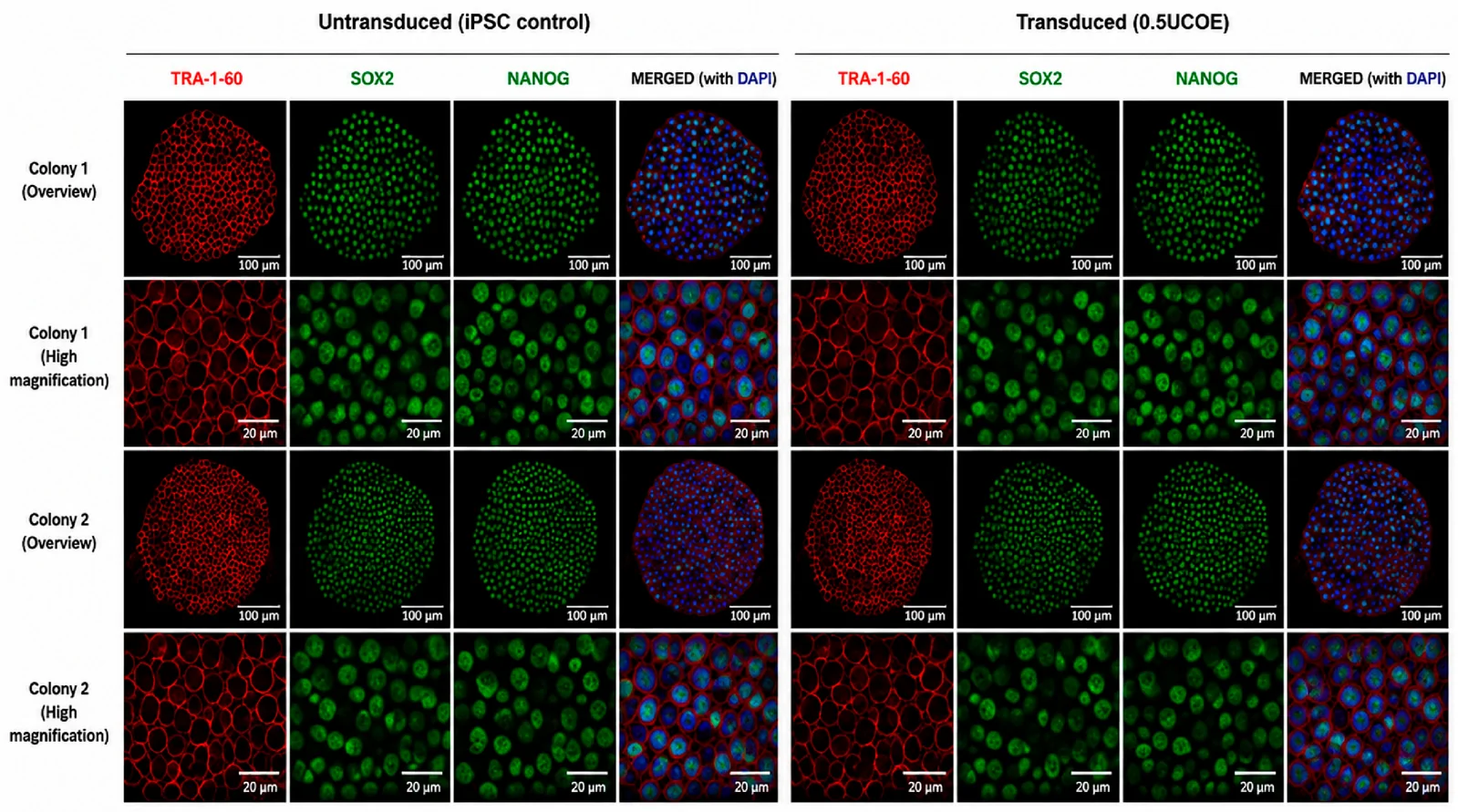
Pluripotency marker expression in untransduced and transduced human iPSC. Pluripotency marker expression in untransduced and transduced human iPSCs. Representative confocal immunofluorescence images of pluripotency markers TRA-1-60, SOX2, and NANOG in untransduced (iPSC control) and lentivirally transduced (0.5UCOE) human iPSC colonies. Nuclei were counterstained with DAPI. Strong expression of all three pluripotency-associated markers was maintained after lentiviral transduction, indicating preservation of the pluripotent phenotype. Scale bars = 100 µm (overview) and 20 µm (high magnification).

The pluripotency-associated phenotype was further evaluated by confocal immunofluorescence staining for TRA-1-60, SOX2, and NANOG. Both untransduced and transduced iPSC cultures exhibited strong immunoreactivity for all three pluripotency-associated markers, with comparable staining patterns between the groups (Figure 5). TRA-1-60 showed the expected predominantly cell-surface distribution, whereas SOX2 and NANOG signals were predominantly nuclear. Representative immunofluorescence images of TRA-1-60, SOX2, and NANOG expression in untransduced and lentivirally transduced human iPSCs are shown in Supplementary Figure S5.

To quantitatively assess potential differences in pluripotency-associated marker expression, fluorescence intensity was measured for TRA-1-60, SOX2, and NANOG using identical image-acquisition and analysis settings for untransduced and transduced cultures. No statistically significant differences were detected between the two groups for any of the three markers.

As an independent molecular assessment, the expression of the pluripotency-associated genes POU5F1 (OCT4), SOX2, and NANOG was further examined by RT-qPCR. Relative transcript levels did not differ significantly between untransduced and transduced iPSCs for any of the three genes.

Collectively, these findings indicate that lentiviral transduction under the experimental conditions used in this study did not produce detectable alterations in iPSC morphology, colony expansion, or pluripotency-associated marker expression at either the protein or transcript level.

### 2.3. Long-Term Stability of Transgene Expression in Undifferentiated Human iPSCs

**Figure 6:**
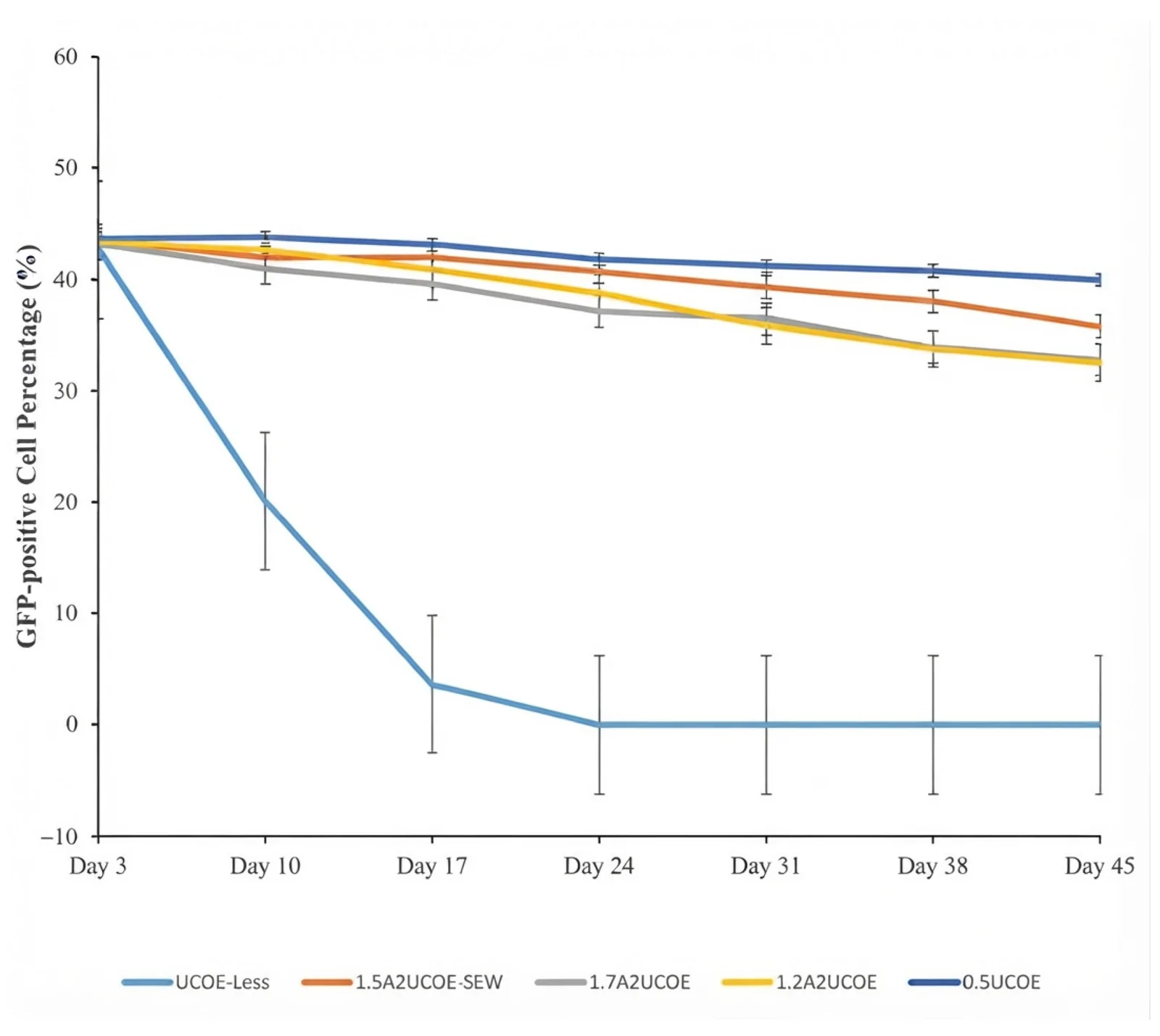
Long-term maintenance of eGFP-positive human iPSCs following lentiviral transduction. Percentage of eGFP-positive cells determined by flow cytometry during 45 days of culture in undifferentiated human iPSCs transduced with UCOE-containing or UCOE-less lentiviral vectors. The UCOE-less control exhibited a progressive decline in reporter-positive cells, whereas vectors containing 1.7 kb, 1.2 kb, or 0.5 kb UCOEs maintained stable transgene expression throughout the observation period. Color coding: UCOE-less (negative control) = orange; A2UCOE-SEW (positive control) = gray; 1.2 kb UCOE = green; 1.7 kb UCOE = purple; 0.5 kb UCOE = dark blue. Data are presented as mean ± SEM (*n* = 3 independent biological replicates). Statistical analysis was performed using two-way repeated-measures ANOVA with construct and time as factors, followed by Tukey’s multiple-comparisons test.

To determine whether the different UCOEs could sustain long-term transgene expression in pluripotent stem cells, eGFP expression was monitored weekly for 45 days following lentiviral transduction. Flow cytometric analysis demonstrated marked differences in the stability of reporter gene expression among the experimental groups (Figure 6).

The UCOE-less control vector exhibited a progressive decline in the percentage of eGFP-positive cells throughout the observation period, indicating gradual transgene silencing in undifferentiated human iPSCs. In contrast, all three UCOE-containing vectors, as well as the A2UCOE-SEW positive control, maintained substantially higher proportions of eGFP-positive cells during the entire 45-day culture period. Among these constructs, the 1.7 kb UCOE consistently demonstrated the highest level of expression stability, whereas both the 1.2 kb and the minimal 0.5 kb UCOE constructs effectively preserved reporter gene expression with only minor reductions over time (Figure 6).

**Figure 7:**
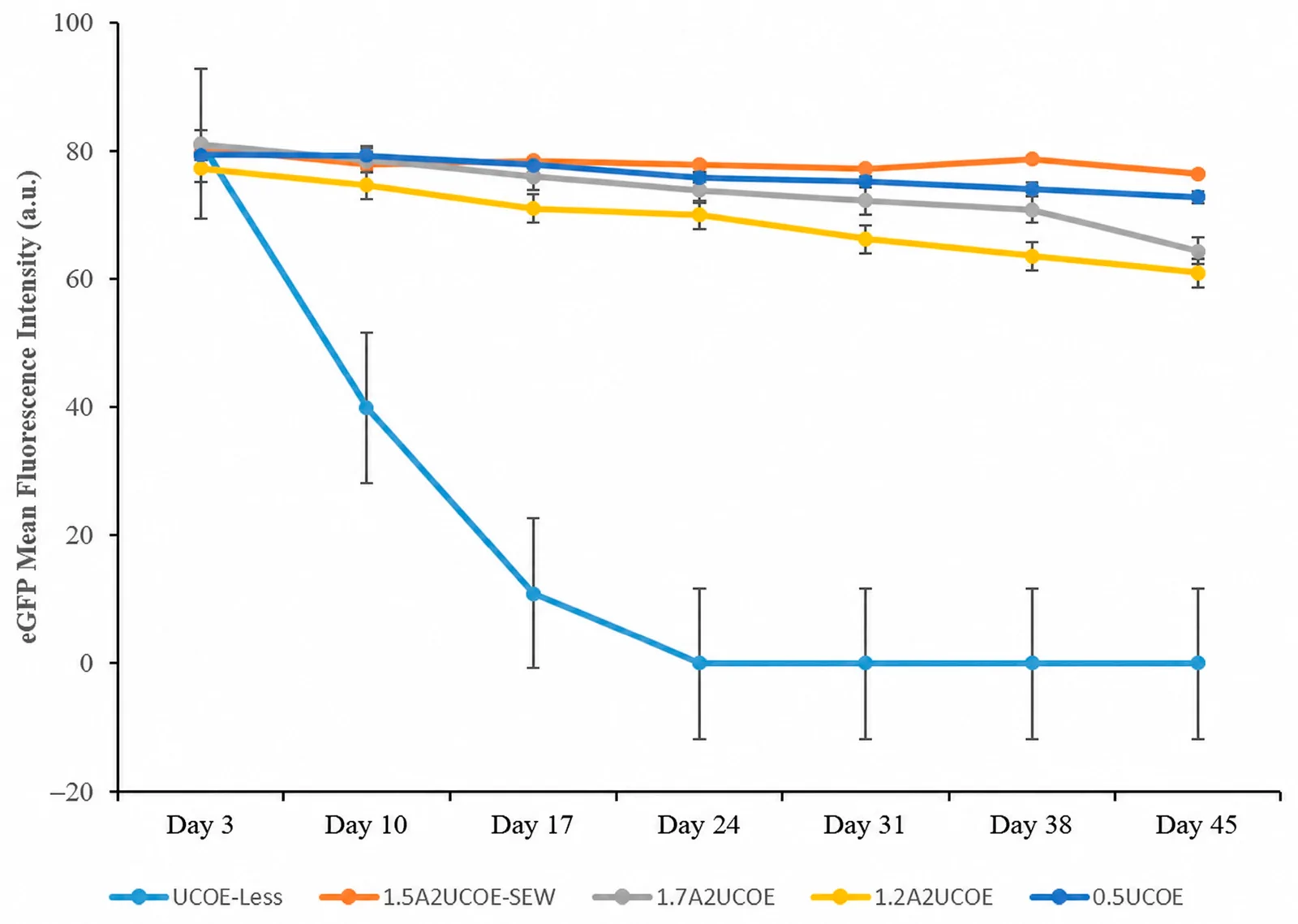
Mean fluorescence intensity of eGFP expression during long-term culture of undifferentiated human iPSCs. Raw eGFP mean fluorescence intensity (MFI) was determined by flow cytometry over a 45-day culture period and is reported in arbitrary fluorescence units (a.u.). MFI was analyzed independently from the percentage of eGFP-positive cells shown in Figure 6. No percentage transformation or normalization to baseline or reference fluorescence was applied to the MFI values. UCOE-containing constructs maintained comparatively stable eGFP fluorescence intensity during prolonged culture, whereas the UCOE-less control showed a progressive decline in detectable reporter fluorescence. Data are presented as mean ± SEM (*n* = 3 independent biological replicates). Statistical analysis was performed using two-way repeated-measures ANOVA with construct and time as factors, followed by Tukey’s multiple-comparisons test.

Changes in reporter gene expression were further evaluated by measuring the mean fluorescence intensity (MFI) of eGFP-positive cells. Consistent with the percentage of transduced cells, the UCOE-less control displayed a continuous reduction in fluorescence intensity, whereas UCOE-containing vectors maintained relatively stable MFI values throughout the experimental period (Figure 7). The 1.7 kb construct produced the highest fluorescence intensity overall, while the 1.2 kb and 0.5 kb constructs exhibited comparable expression profiles, indicating sustained transcriptional activity despite their reduced UCOE sizes.

**Figure 8:**
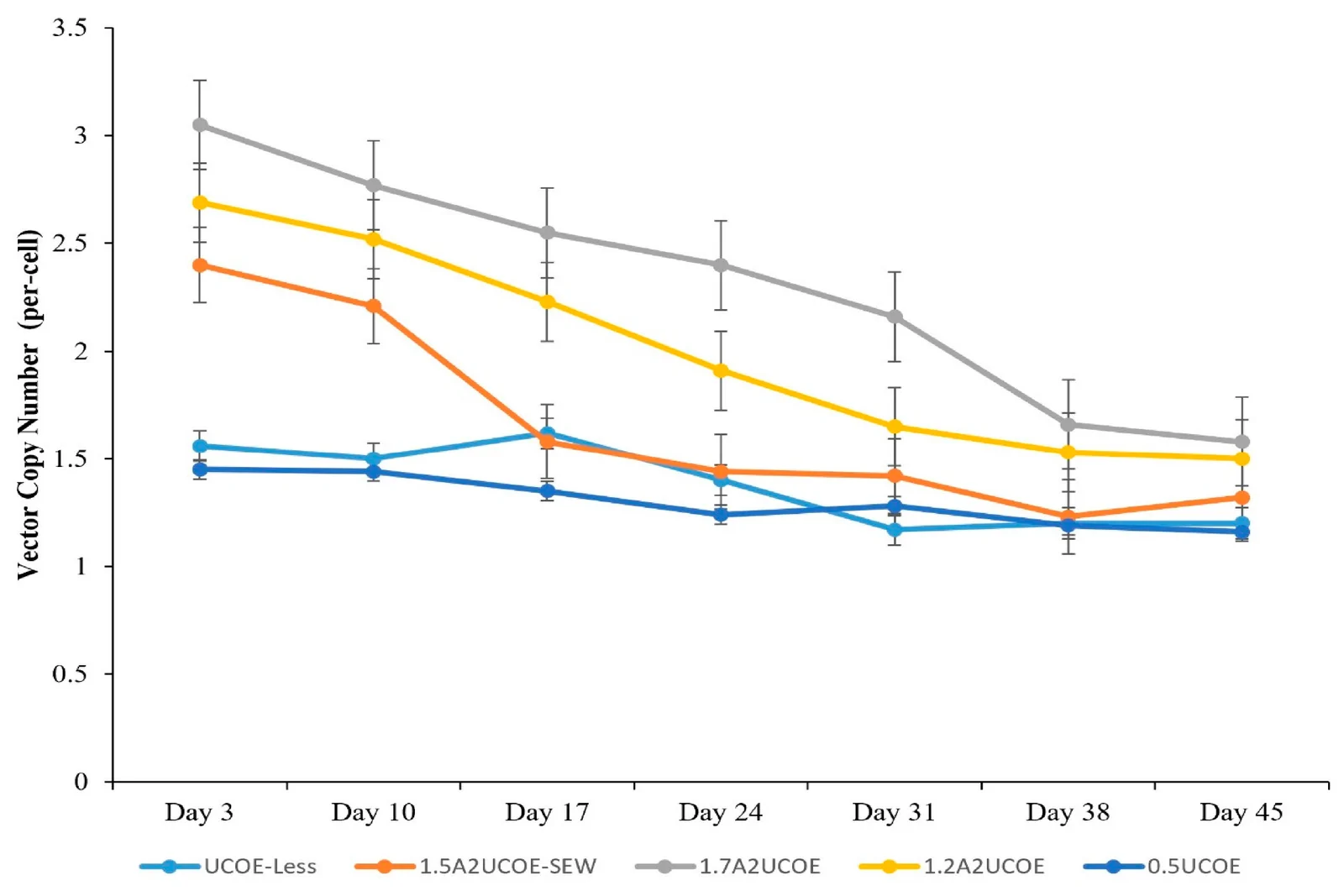
Stability of integrated lentiviral vector copy number during long-term culture. Vector copy number (VCN) was determined by quantitative PCR in undifferentiated human iPSCs transduced with the indicated lentiviral vectors during the 45-day experimental period. Color coding: UCOE-less (negative control) = orange; A2UCOE-SEW (positive control) = gray; 1.2 kb UCOE = green; 1.7 kb UCOE = purple; 0.5 kb UCOE = dark blue. Individual symbols represent independent biological replicates (*n* = 3). Where the same biological replicate was followed longitudinally, corresponding measurements are connected across time. Data are presented as mean ± SEM. Statistical analysis was performed using two-way repeated-measures ANOVA with construct and time as factors, followed by Tukey’s multiple-comparisons test. No significant construct effect was detected among the UCOE-containing groups over the observation period. This finding indicates comparable VCN levels among constructs but does not imply complete longitudinal stability within individual constructs.

To distinguish transcriptional silencing from potential changes in integrated lentiviral vector abundance, vector copy number (VCN) was quantified by qPCR at the designated experimental time points. Longitudinal VCN measurements showed that the UCOE-containing constructs remained detectable throughout the 45-day culture period, although the magnitude and direction of temporal changes varied among constructs (Figure 8). Importantly, the absence of a statistically significant difference among the UCOE-containing constructs should not be interpreted as evidence of complete longitudinal VCN stability within each individual construct. Rather, the data indicate that the UCOE-containing groups maintained comparable levels of detectable vector copies over the observation period. These findings further indicate that sustained eGFP expression in the UCOE-containing groups cannot be attributed simply to invariant vector copy number, but occurred despite longitudinal variation in VCN, consistent with a role for UCOEs in maintaining transcriptional competence of the integrated expression cassette.

Collectively, these results demonstrate that inclusion of UCOEs substantially improves the long-term stability of transgene expression in human iPSCs. Notably, the newly designed 0.5 kb minimal UCOE displayed expression stability comparable to the larger UCOE constructs, suggesting that reduction in UCOE size can be achieved without markedly compromising its anti-silencing activity.

Representative primary flow-cytometry plots and the corresponding gating strategy underlying the longitudinal eGFP-positive cell percentage and MFI measurements are provided in Supplementary Figure S2.

### 2.4. Neuronal Differentiation of UCOE-Transduced Human iPSCs

Following long-term evaluation of transgene stability in the undifferentiated state, human iPSCs transduced with the different UCOE-containing lentiviral vectors were subjected to neuronal differentiation in order to determine whether stable transgene expression could be maintained during lineage commitment and neuronal maturation.

**Figure 9:**
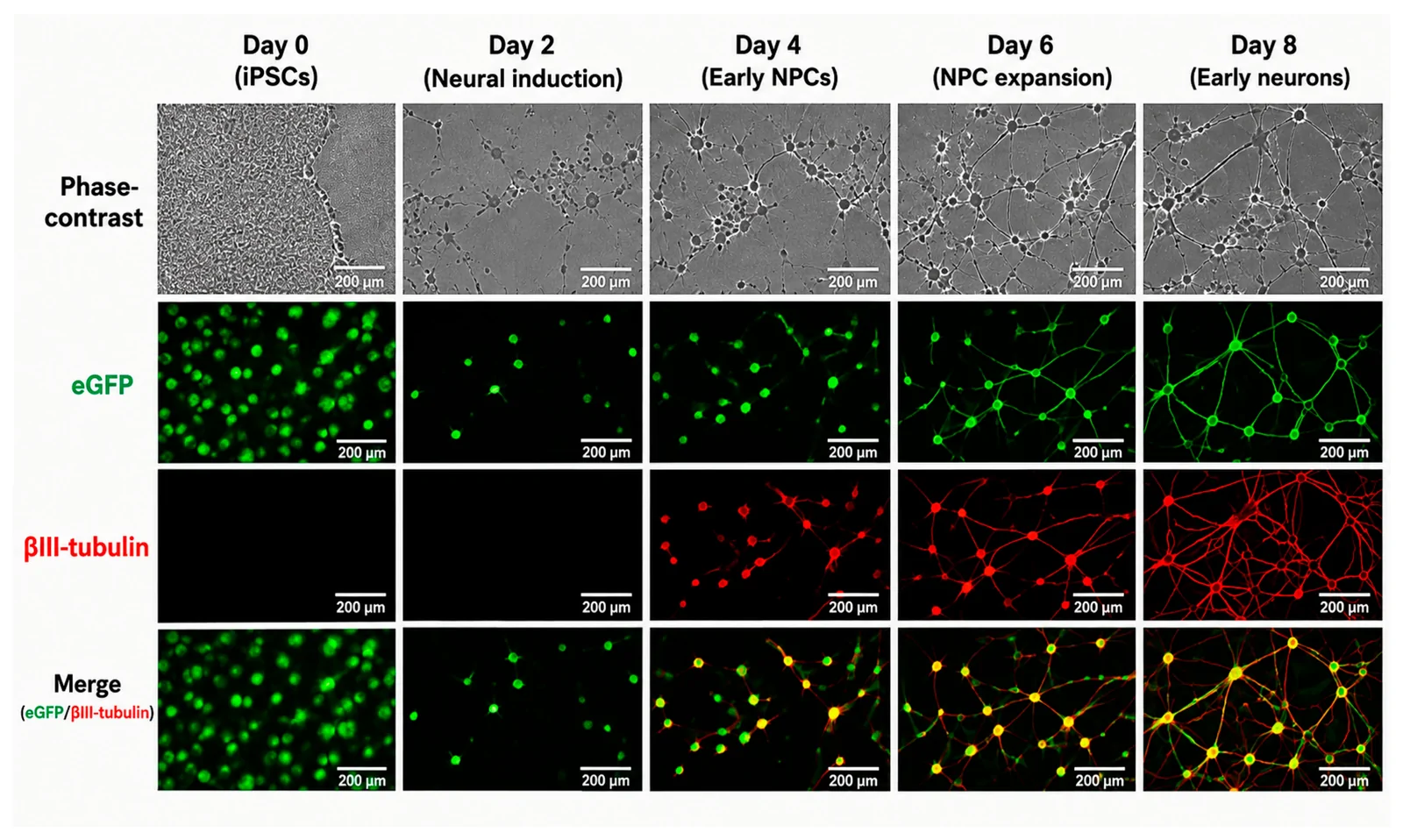
Neural differentiation of UCOE-transduced human iPSCs during early lineage commitment. Representative phase-contrast and fluorescence images illustrating sequential morphological and phenotypic changes during the early stages of neural differentiation. The indicated stages were operationally defined according to the timing of the differentiation protocol, characteristic morphological changes, and neural lineage-associated marker expression where assessed. Day 0 represents undifferentiated iPSCs displaying compact cellular organization. Day 2 (neural induction) corresponds to the initial morphological transition following exposure to neural induction conditions. Day 4 (early NPC-like stage) is characterized by the emergence of cells displaying neural progenitor-like morphology. Day 6 (NPC expansion) shows further expansion and organization of cells with neural progenitor-like morphology. Day 8 (early neuronal stage) is characterized by increasingly elongated neuronal-like cells, prominent neuritic extensions, interconnected cellular networks, and βIII-tubulin (TUJ1) immunoreactivity. Intrinsic eGFP fluorescence was monitored throughout differentiation to assess persistence of reporter expression. Merge images show the spatial relationship between eGFP fluorescence (green) and βIII-tubulin immunoreactivity (red), where applicable. These stage designations are operational descriptions of the temporal progression of the in vitro differentiation protocol and do not imply homogeneous or completely stage-restricted cell populations. Scale bars = 200 µm for all panels.

During early neural differentiation, UCOE-transduced iPSCs underwent progressive morphological changes consistent with neural lineage commitment (Figure 9). Undifferentiated cells at Day 0 exhibited the compact organization characteristic of iPSC cultures. Following neural induction, an initial change in cellular morphology was apparent by Day 2. By Day 4, cells increasingly displayed neural progenitor-like morphology and organization, accompanied by the emergence of βIII-tubulin immunoreactivity. Further cellular expansion and development of βIII-tubulin-positive processes were observed by Day 6. By Day 8, the cultures contained increasingly elongated neuronal-like cells with prominent neuritic extensions and interconnected networks, together with more extensive βIII-tubulin immunoreactivity.

**Figure 10:**
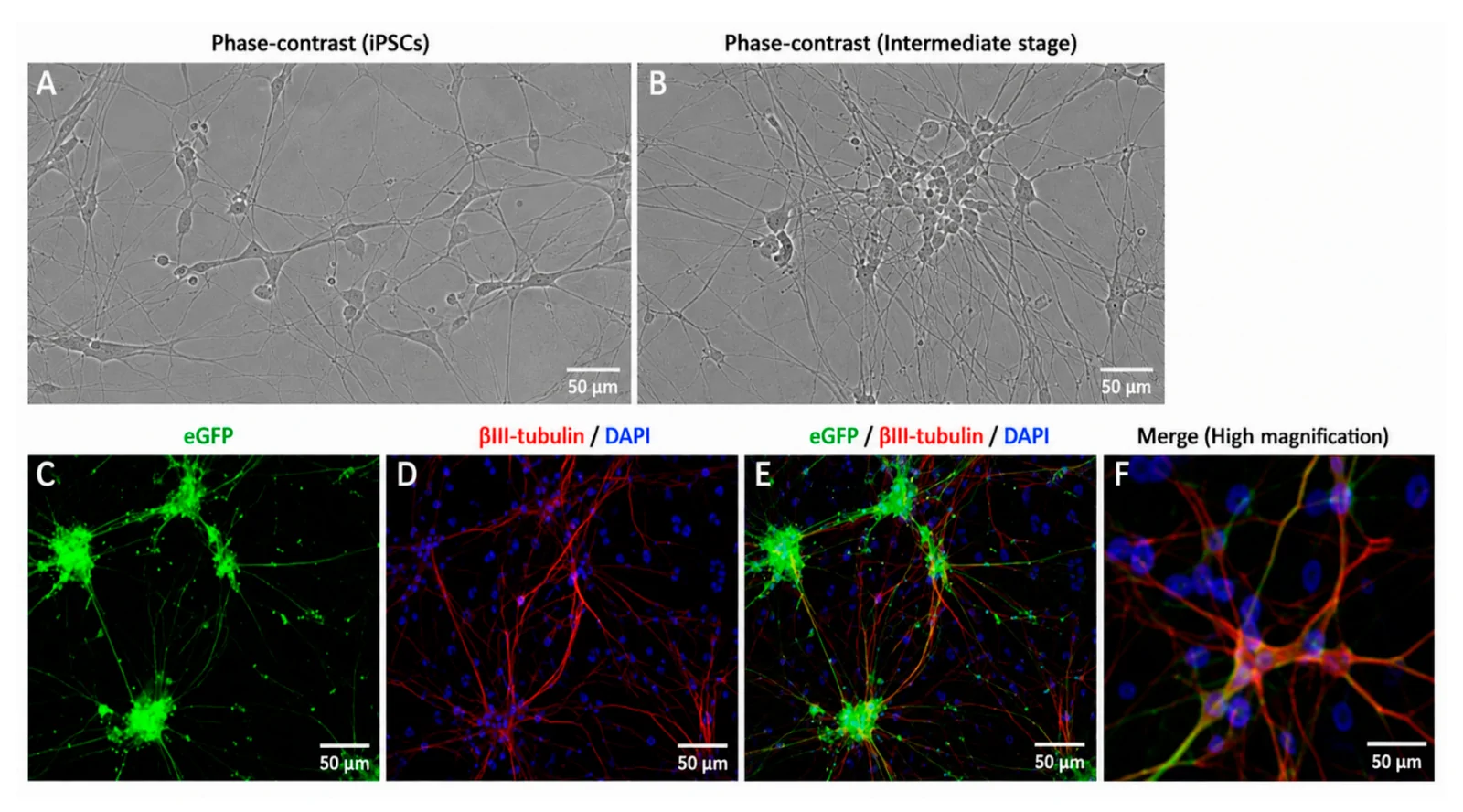
Co-expression of eGFP and βIII-tubulin in iPSC-derived neurons. Representative images of differentiated cultures showing morphological progression and neuronal marker expression. (**A**) Phase-contrast image of iPSCs during the differentiation process. (**B**) Phase-contrast image of cells at the intermediate stage of neuronal differentiation. (**C**) eGFP fluorescence showing reporter-positive cells and neurite-associated fluorescence. (**D**) βIII-tubulin (TUJ1) immunofluorescence with DAPI nuclear counterstaining, demonstrating neuronal marker expression. (**E**) Merged eGFP/βIII-tubulin/DAPI image showing co-localization of reporter fluorescence with βIII-tubulin-positive neuronal cells. (**F**) High-magnification merged image illustrating eGFP and βIII-tubulin fluorescence in differentiated neuronal cells. Representative βIII-tubulin-positive cells exhibiting detectable eGFP fluorescence demonstrate persistence of reporter expression following neuronal commitment. eGFP/βIII-tubulin co-expression indicates reporter persistence in differentiated transduced cells and is not intended as a measure of neuronal differentiation efficiency. Scale bars = 50 µm.

As differentiation progressed, neuronal maturation was accompanied by extensive neurite extension and the establishment of complex neuronal networks. Immunofluorescence analysis demonstrated robust expression of the neuronal marker βIII-tubulin (TUJ1), confirming successful neuronal differentiation. Confocal microscopy demonstrated βIII-tubulin-positive cells exhibiting detectable eGFP fluorescence, indicating that reporter expression was retained in a subset of differentiated neuronal cells following neuronal commitment (Figure 10). Importantly, neuronal marker expression was not restricted to cells exhibiting detectable eGFP fluorescence. Examination of the individual fluorescence channels and merged confocal images demonstrated the presence of βIII-tubulin-positive cells without detectable eGFP fluorescence within the differentiated cultures. Thus, the observed co-expression of eGFP and neuronal markers represents persistence of reporter expression in a subset of differentiated transduced cells and should not be interpreted as evidence that eGFP-positive cells preferentially underwent neuronal differentiation. The present experiments were designed to evaluate maintenance of reporter expression during neuronal differentiation rather than to quantitatively compare neuronal differentiation efficiency between eGFP-positive and eGFP-negative populations.

**Figure 11:**
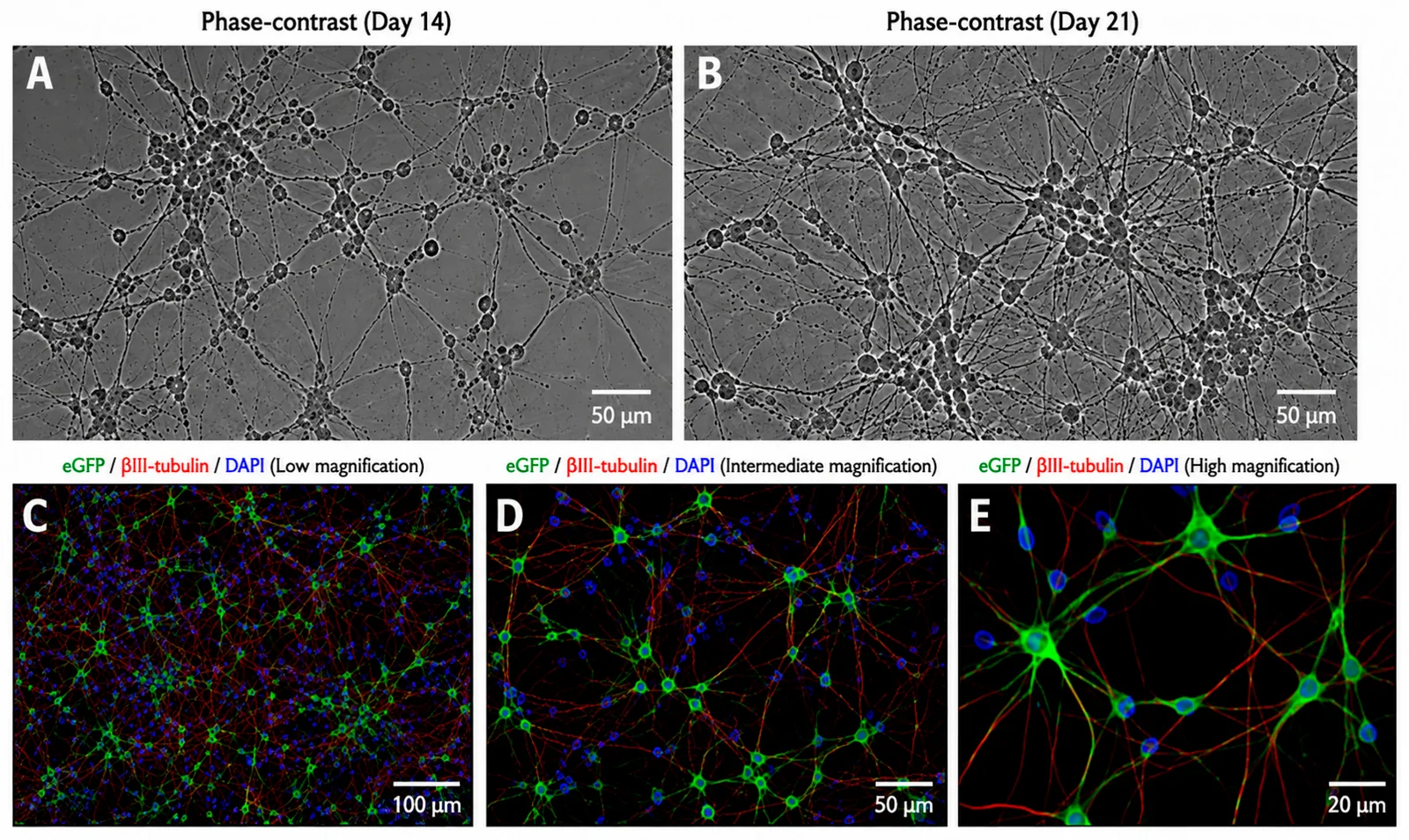
Morphological and fluorescence characterization of later-stage iPSC-derived neuronal networks with sustained reporter expression. Representative phase-contrast and fluorescence/confocal microscopy images of differentiated neuronal cultures at Days 14 and 21. (**A**) Phase-contrast image of the differentiated neuronal culture at Day 14, showing extensive neurite extension and developing interconnected neuronal networks. (**B**) Phase-contrast image of the differentiated neuronal culture at Day 21, demonstrating increased structural complexity and extensive interconnected neuronal networks. (**C**) Low-magnification merged fluorescence image showing eGFP reporter expression (green), βIII-tubulin immunoreactivity (red), and DAPI nuclear counterstaining (blue) throughout the differentiated neuronal culture. (**D**) Intermediate-magnification merged fluorescence image showing eGFP-positive cells and βIII-tubulin-positive neuronal processes with DAPI nuclear counterstaining. (**E**) High-magnification merged fluorescence image illustrating eGFP and βIII-tubulin fluorescence in differentiated neuronal cells and their neurite structures. Scale bars: 50 µm for (**A**,**B**), 100 µm for (**C**), 50 µm for (**D**), and 20 µm for (**E**). βIII-tubulin-positive cells without detectable eGFP fluorescence were also observed, indicating that eGFP/βIII-tubulin co-expression reflects reporter persistence rather than preferential neuronal differentiation of eGFP-positive cells.

Based on the timing of the differentiation protocol, characteristic morphological changes, and the appearance of neural lineage-associated marker expression where assessed, these time points were operationally designated as neural induction (Day 2), early NPC-like stage (Day 4), NPC expansion (Day 6), and early neuronal stage (Day 8). These designations represent operational stages of the in vitro differentiation process and do not imply homogeneous or stage-restricted cell populations. Intrinsic eGFP fluorescence remained detectable throughout these morphological transitions, supporting persistence of reporter expression during early neural differentiation. Days 14–21 were operationally designated as the mature neuronal network stage based on extensive neurite branching, increased structural complexity, and the formation of interconnected neuronal networks (Figure 11). These stage designations represent operational descriptions of the temporal progression of the in vitro differentiation process and do not imply homogeneous or completely stage-restricted cell populations.

Continued differentiation resulted in neuronal cells displaying highly branched neuritic processes and increased structural complexity. At these later stages, the cultures exhibited morphological features consistent with neuronal maturation, while eGFP fluorescence remained detectable throughout neuronal cell bodies and processes (Figure 11). These observations support persistence of reporter expression during morphological neuronal maturation but do not establish electrophysiological or synaptic maturity.

**Figure 12:**
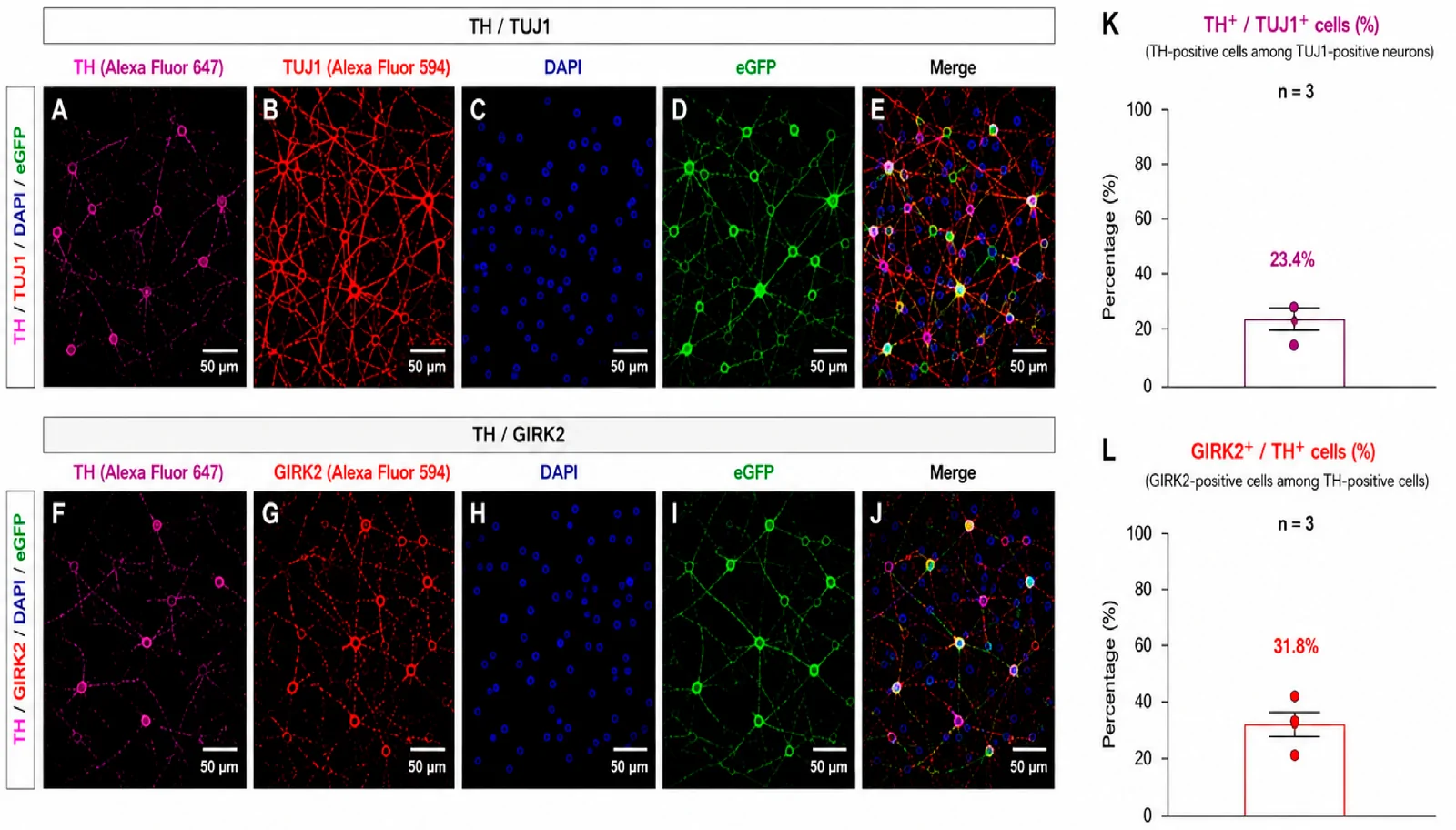
Neuronal and catecholaminergic characterization of UCOE-transduced iPSC-derived neurons. Representative confocal immunofluorescence images and quantitative analysis of neuronal and catecholaminergic-associated marker expression in differentiated neuronal cultures. (**A**–**E**) TH immunoreactivity detected using an Alexa Fluor 647-conjugated secondary antibody (displayed in magenta), βIII-tubulin (TUJ1) immunoreactivity detected using an Alexa Fluor 594-conjugated secondary antibody (red), DAPI nuclear counterstaining (blue), intrinsic eGFP fluorescence (green), and the corresponding merged image, respectively. (**F**–**J**) TH immunoreactivity (Alexa Fluor 647; magenta), GIRK2 immunoreactivity (Alexa Fluor 594; red), DAPI (blue), intrinsic eGFP fluorescence (green), and the corresponding merged image, respectively. TH immunoreactivity and intrinsic eGFP fluorescence were acquired independently in spectrally distinct fluorescence channels. (**K**) Quantification of TH-positive cells within the TUJ1-positive neuronal population (TH^+^/TUJ1^+^). (**L**) Quantification of GIRK2-positive cells within the TH-positive population (GIRK2^+^/TH^+^). Quantitative analyses were performed using three independent biological replicates (*n* = 3); individual symbols represent biological replicates and summary values are presented as mean ± SEM. TH-positive cells represented 23.4% of the TUJ1-positive neuronal population, whereas 31.8% of TH-positive cells were GIRK2-positive, demonstrating that TH- and GIRK2-immunoreactive cells constituted subpopulations within the heterogeneous differentiated neuronal cultures rather than the majority of the neuronal population. Because the differentiation protocol did not include ventral midbrain patterning factors, TH and GIRK2 expression is interpreted as phenotypic characterization of catecholaminergic-associated neuronal subpopulations and not as evidence of directed dopaminergic specification. TH-, TUJ1-, and GIRK2-immunoreactive cells were not restricted to cells exhibiting detectable eGFP fluorescence; therefore, marker/eGFP co-expression is interpreted as evidence of reporter persistence within differentiated transduced cells rather than preferential neuronal differentiation of the eGFP-positive population. Scale bars = 50 µm for panels (**A**–**J**).

To further characterize the differentiated neuronal population, mature cultures were immunostained for the neuronal marker βIII-tubulin (TUJ1) and the catecholaminergic-associated markers tyrosine hydroxylase (TH) and G protein-activated inward rectifier potassium channel 2 (GIRK2). Quantitative analysis of three independent biological replicates demonstrated that TH-positive cells represented 23.4 ± 2.1% of the TUJ1-positive neuronal population. Among the TH-positive cells, 31.8 ± 3.4% also exhibited GIRK2 immunoreactivity. Individual biological replicate values are presented in Figure 12K,L. These quantitative findings demonstrate that the differentiated cultures were heterogeneous and that neither the TH^+^/TUJ1^+^ nor GIRK2^+^/TH^+^ population approached 100%. Accordingly, TH and GIRK2 expression was interpreted as phenotypic characterization of neuronal subpopulations rather than evidence of highly efficient or directed dopaminergic specification. The differentiation protocol employed here was a general neuronal induction protocol and did not include ventral midbrain patterning factors. Importantly, neuronal marker expressions were also observed in cells without detectable eGFP fluorescence, indicating that the observed marker/eGFP co-expression reflects reporter persistence in a subset of differentiated transduced cells rather than preferential neuronal differentiation of eGFP-positive cells.

Collectively, these findings demonstrate that lentivirally delivered UCOEs support sustained transgene expression throughout neuronal differentiation and maturation. The imaging data further indicate that neuronal marker expression was not restricted to cells with detectable eGFP fluorescence. Because neuronal differentiation efficiency was not quantitatively compared between eGFP-positive and eGFP-negative populations, these experiments do not establish an effect of the UCOE-containing vectors on neuronal differentiation itself. Importantly, stable reporter expression was preserved from the pluripotent stage through terminal neuronal differentiation, highlighting the utility of these UCOE constructs for long-term gene expression in stem cell-based applications.

### 2.5. Long-Term Transgene Stability During Neuronal Differentiation

Following confirmation of successful neuronal differentiation, long-term transgene stability was quantitatively assessed throughout both the undifferentiated culture period and the subsequent neuronal differentiation stage. In this arm of the study, neuronal differentiation was initiated on Day 5 post-transduction and eGFP expression was monitored weekly by flow cytometry from Day 3 to Day 60, thereby covering the short undifferentiated interval before induction as well as the entire differentiation and maturation period. This arm is distinct from the 45-day undifferentiated arm reported in Section 2.3, in which parallel cultures were maintained in the pluripotent state without induction.

**Figure 13:**
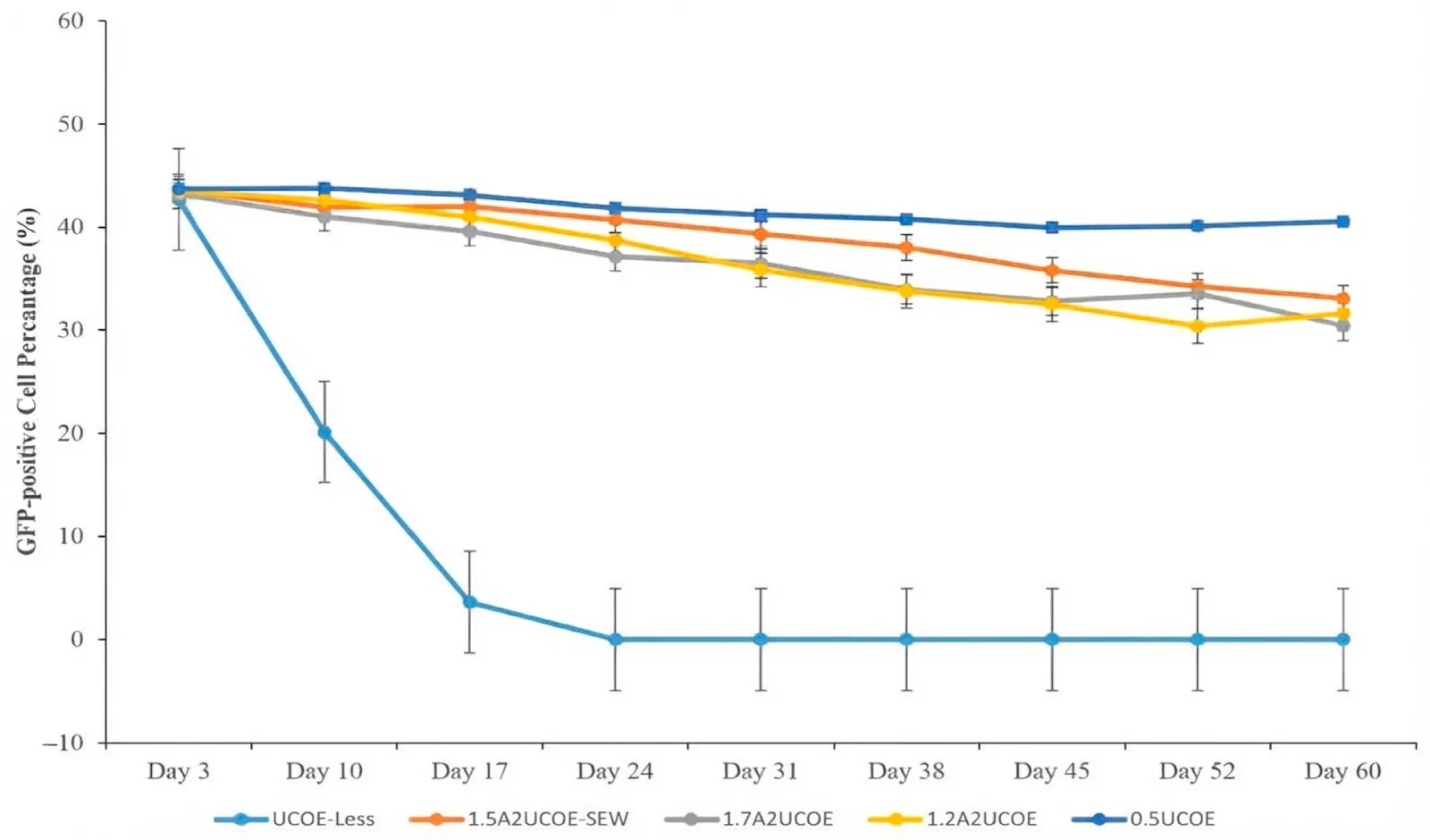
Longitudinal flow cytometric analysis of eGFP-positive cells following lentiviral transduction of human iPSCs. Weekly percentages of eGFP-positive cells were quantified from day 3 to day 60, including both the undifferentiated maintenance phase and neuronal differentiation. The UCOE-less construct exhibited progressive loss of reporter expression, whereas all UCOE-containing vectors maintained stable eGFP positivity throughout the experimental period. Color coding: UCOE-less (negative control) = orange; A2UCOE-SEW (positive control) = gray; 1.2 kb UCOE = green; 1.7 kb UCOE = purple; 0.5 kb UCOE = dark blue. Data are presented as mean ± SEM (*n* = 4; *p* < 0.01).

As shown in Figure 13, the UCOE-less vector exhibited a continuous decline in the percentage of eGFP-positive cells throughout the experiment, indicating progressive transgene silencing during prolonged culture and neuronal differentiation. In contrast, all UCOE-containing vectors, together with the A2UCOE-SEW positive control, maintained substantially higher percentages of eGFP-positive cells over the entire experimental period. Among the experimental constructs, the 1.7 kb UCOE exhibited the highest overall level of transgene expression, whereas the newly developed 0.5 kb UCOE demonstrated an expression profile comparable to those of the 1.2 kb and 1.7 kb UCOE constructs despite its substantially reduced size. Longitudinal VCN analysis showed that vector copies remained detectable throughout the 60-day experimental period, although temporal variation in VCN was observed among the individual constructs. The longitudinal changes in the proportion of eGFP-positive human iPSCs throughout the 60-day experimental period are shown in Supplementary Figure S4.

**Figure 14:**
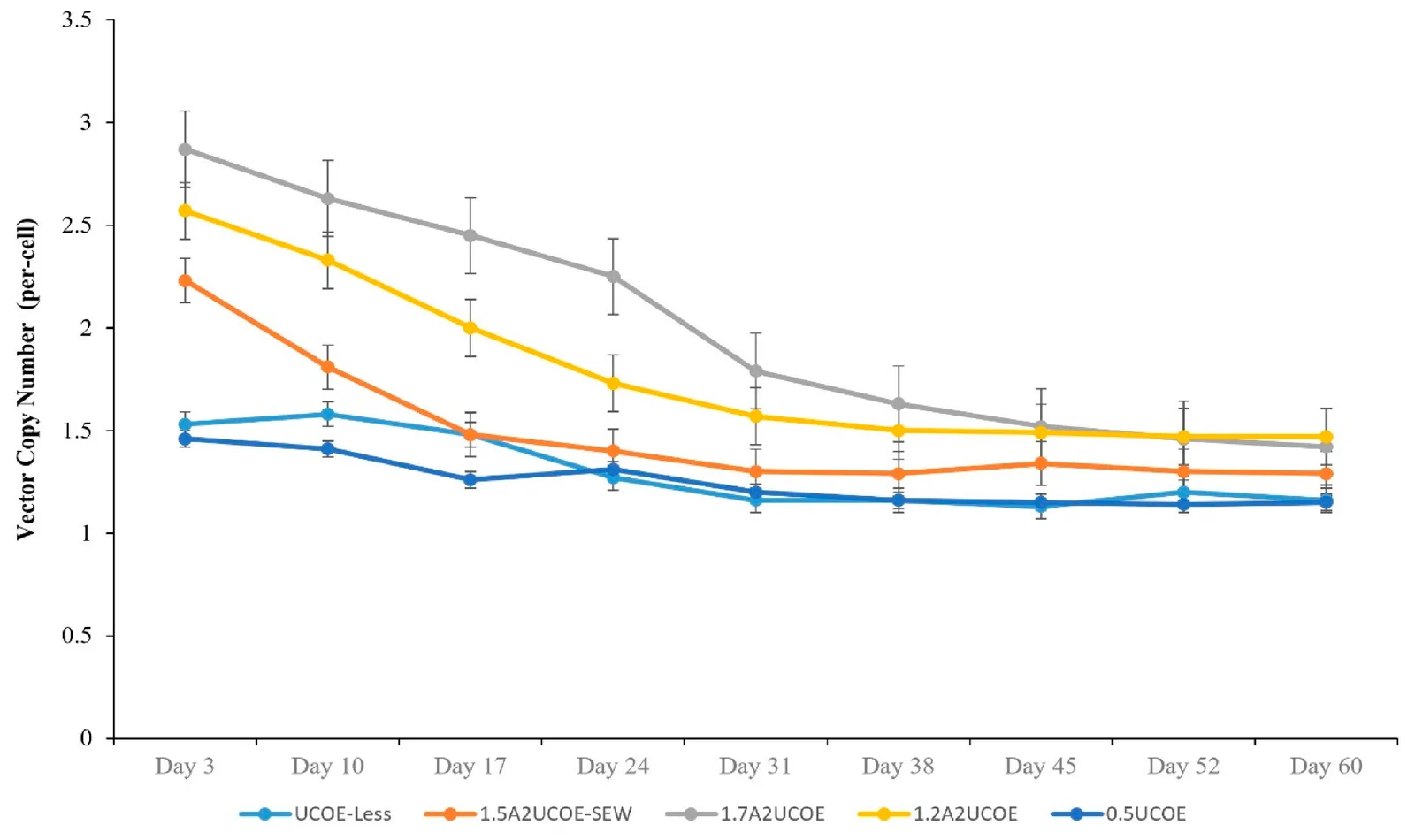
Longitudinal analysis of vector copy number following lentiviral transduction. Vector copy number was determined weekly by qPCR throughout the 60-day experimental period. UCOE-containing vectors maintained relatively stable genomic vector copy numbers during undifferentiated culture and neuronal differentiation. Color coding: UCOE-less = light blue; 1.5A2UCOE-SEW = orange; 1.7A2UCOE = gray; 1.2A2UCOE = yellow; 0.5UCOE = dark blue. Individual symbols represent independent biological replicates (*n* = 4). Where the same biological replicate was followed longitudinally, corresponding measurements are connected across time. Data are presented as mean ± SEM.

To determine whether the observed changes in reporter expression reflected transcriptional silencing rather than loss of integrated lentiviral vectors, vector copy number (VCN) was quantified weekly by qPCR. As illustrated in Figure 14, vector copies remained detectable in all UCOE-containing groups throughout both undifferentiated culture and neuronal differentiation, although longitudinal variation in VCN was observed over the experimental period. Although detectable eGFP expression in the UCOE-less group progressively declined during prolonged culture, these cells were maintained throughout the complete 60-day experimental period and were included in the longitudinal VCN analysis. Accordingly, VCN measurements for the UCOE-less group are presented through Day 60 in Figure 14. Because detectable eGFP expression became very low after Day 31, later time points were not included in the comparative expression analysis. The persistence of detectable vector copies despite the loss of reporter fluorescence is consistent with transcriptional silencing rather than complete physical loss of the integrated vector.

Overall, the combined flow cytometric and vector copy number analyses demonstrate that UCOE-containing lentiviral vectors support sustained transgene expression during prolonged culture and neuronal differentiation. Importantly, the minimized 0.5 kb UCOE performed comparably to the larger 1.2 kb and 1.7 kb constructs, confirming that substantial reduction in the UCOE sequence did not impair its ability to preserve long-term transcriptional activity.

The percentage of eGFP-positive cells progressively declined in the UCOE-less group, whereas the UCOE-containing constructs maintained detectable reporter expression throughout the experimental period. Representative flow-cytometric profiles of long-term eGFP expression during the 45-day undifferentiated culture period are shown in Supplementary Figure S3. eGFP MFI values showed a similar pattern, with substantially higher fluorescence intensity maintained in the UCOE-containing groups compared with the UCOE-less group. Representative primary flow-cytometry plots and the corresponding gating strategy underlying the longitudinal eGFP-positive cell percentage and MFI measurements are provided in Supplementary Figure S2.

## 3. Discussion

The maintenance of stable transgene expression remains one of the major challenges in gene therapy, particularly in pluripotent stem cells, where extensive epigenetic remodeling frequently results in progressive transcriptional silencing of integrated viral vectors. Lentiviral vectors provide efficient and stable genomic integration; however, their therapeutic efficacy is often compromised by promoter DNA methylation, heterochromatin formation, histone modifications, and position-effect variegation following chromosomal integration [3,13]. Ubiquitous chromatin opening elements (UCOEs), originally identified within the divergent promoter region of the HNRPA2B1–CBX3 locus, establish and maintain an open chromatin environment that protects integrated transgenes from epigenetic repression and supports sustained transcriptional activity [6,7,24]. Consequently, UCOEs have emerged as one of the most promising cis-regulatory elements for achieving long-term and predictable transgene expression in stem cell-based gene therapy [2,23].

In the present study, we systematically compared three UCOE constructs of different sizes (1.7 kb, 1.2 kb, and a newly designed 0.5 kb minimal UCOE) in human induced pluripotent stem cells and during neuronal differentiation. Our results demonstrate that incorporation of UCOE sequences markedly improved long-term reporter gene expression compared with the UCOE-less control. Whereas progressive reduction in eGFP expression was observed in the absence of UCOE sequences, all three UCOE-containing vectors maintained stable reporter expression throughout the 45-day observation period. Importantly, vector copy number remained relatively constant during culture, indicating that the reduction in reporter expression observed in the control group resulted primarily from transcriptional silencing rather than the physical loss of integrated lentiviral genomes. These observations are consistent with previous studies demonstrating that UCOEs primarily preserve transcriptionally permissive chromatin without significantly altering vector integration efficiency or genomic copy number [2,17,24].

One of the principal findings of the present study is that the newly designed 0.5 kb UCOE retained substantial anti-silencing activity despite its markedly reduced size. Previous investigations have demonstrated that the classical A2UCOE fragment (approximately 1.5–2.2 kb) effectively maintains long-term transgene expression in hematopoietic stem cells, embryonic stem cells, induced pluripotent stem cells, and various differentiated cell types [2,17]. Nevertheless, the relatively large size of conventional UCOEs occupies valuable vector capacity, thereby limiting the incorporation of large therapeutic transgenes or complex regulatory cassettes. Our findings suggest that the essential chromatin-opening properties of A2UCOE may be retained within a substantially smaller functional region, thereby providing additional packaging capacity without markedly compromising anti-silencing activity.

The comparable performance of the minimal 0.5 kb construct is consistent with the proposed anti-silencing properties of UCOEs, which have previously been associated with maintenance of a transcriptionally permissive chromatin environment and resistance to epigenetic repression [6,7,19,24]. However, a limitation of the present study is that the molecular epigenetic mechanisms underlying the observed anti-silencing phenotype were not directly examined. Although the sustained reporter expression and relatively stable vector copy number are consistent with preservation of transcriptional competence rather than loss of integrated vector genomes, DNA methylation, chromatin accessibility, and histone modification status were not measured in the present experimental framework. Therefore, the observed phenotype should not be interpreted as direct evidence of chromatin opening or prevention of heterochromatin formation. Future studies incorporating targeted DNA methylation profiling, chromatin accessibility assays, and histone modification analyses will be required to define the molecular basis of the 0.5-kb UCOE activity.

Since the discovery of the A2UCOE locus by Antoniou and colleagues, UCOEs have been extensively investigated as cis-regulatory elements capable of protecting integrated transgenes from epigenetic silencing in a wide range of mammalian cell types. The original A2UCOE, derived from the divergent promoter region between the HNRPA2B1 and CBX3 housekeeping genes, was shown to maintain long-term transgene expression independently of the integration site by preserving an open chromatin configuration and resisting DNA methylation [6,26]. Subsequent studies demonstrated that this anti-silencing activity extends to hematopoietic stem cells, embryonic stem cells, induced pluripotent stem cells, and mesenchymal stem cells, indicating that UCOE function is largely independent of cell lineage [2,17]. The sustained eGFP expression observed throughout the 45-day culture period in the present study is consistent with these previous reports and supports the functional anti-silencing activity of UCOE-containing lentiviral constructs in human iPSCs.

One of the most significant advances of the present study is the direct comparison of three UCOE fragments differing substantially in length within the same experimental platform. Previous studies have primarily focused on classical A2UCOE fragments ranging between approximately 1.5 and 2.2 kb, and relatively few have systematically evaluated minimal UCOE constructs under identical conditions while assessing long-term transgene stability during neuronal differentiation [6,11,24]. Our results demonstrate that the newly designed 0.5 kb UCOE maintained long-term reporter gene expression with a performance comparable to that observed for the larger 1.2 kb and 1.7 kb constructs. These findings suggest that substantial UCOE minimization can be achieved without a marked loss of functional activity under the experimental conditions examined in this study. From a vector engineering perspective, every kilobase saved within a lentiviral construct may provide additional space for therapeutic genes, regulatory sequences, or other functional elements within the vector cassette without exceeding packaging limitations [7,25], and the present findings provide experimental evidence that substantial UCOE sequence minimization can be achieved while retaining comparable anti-silencing performance under the conditions tested.

An additional strength of the present work is the evaluation of UCOE function throughout neuronal differentiation. Most previous investigations have examined UCOE activity predominantly in proliferating stem cells or immortalized cell lines, whereas relatively few studies have investigated its performance during lineage-specific neuronal differentiation [2,17,24]. Neuronal differentiation is accompanied by extensive epigenetic remodeling, including dynamic changes in DNA methylation, histone modifications, and chromatin accessibility, all of which may contribute to progressive transgene silencing after lineage commitment [3,8,21]. In the present study, stable eGFP expression was maintained not only in undifferentiated iPSCs but also throughout neuronal differentiation and maturation, indicating that UCOE-mediated chromatin protection remains effective despite profound changes in cellular identity. These findings further support the potential utility of minimal UCOE constructs for stem cell engineering, neurological disease modeling, regenerative medicine, and future gene therapy applications.

Importantly, the immunofluorescence findings should be interpreted in the context of the primary objective of this study. Although eGFP-positive cells expressing βIII-tubulin, TH, or GIRK2 were readily observed, neuronal marker-positive cells without detectable eGFP fluorescence were also present in the differentiated cultures. Therefore, the presence of neuronal marker/eGFP co-expression supports persistence of reporter expression in differentiated transduced cells but does not indicate preferential neuronal differentiation of eGFP-positive cells. Because neuronal differentiation efficiency was not quantitatively assessed as a function of eGFP status, the present study does not allow conclusions regarding whether lentiviral transduction or UCOE incorporation affects neuronal differentiation efficiency.

Interestingly, vector copy number analysis demonstrated that integrated lentiviral genomes remained relatively constant throughout long-term culture. Consequently, the gradual decline in reporter expression observed in the UCOE-less control cannot be attributed primarily to vector loss but is more consistent with progressive epigenetic repression of integrated transgenes. Similar observations have been reported in previous mechanistic studies demonstrating that UCOEs preserve long-term transcriptional competence without significantly affecting lentiviral integration frequency or vector copy number [2,17,24]. Therefore, our findings are consistent with the established model that UCOEs primarily exert their anti-silencing effects at the level of transcriptional regulation rather than by increasing vector integration efficiency. However, the specific chromatin and epigenetic mechanisms underlying this effect were not directly assessed in the present study.

Although the precise molecular mechanisms responsible for UCOE-mediated protection against transgene silencing remain incompletely understood, accumulating evidence suggests that these elements establish and maintain a transcriptionally permissive chromatin environment surrounding integrated transgenes. Unlike conventional enhancer elements, UCOEs do not primarily function by increasing promoter strength; rather, they preserve an open chromatin configuration and protect adjacent promoters from progressive heterochromatin formation through epigenetic regulation [6,7,24]. This unique property is particularly advantageous for long-term gene expression because it confers resistance to the progressive epigenetic repression that frequently accompanies lentiviral integration [3,13].

One of the defining characteristics of UCOEs is their origin from CpG island-associated housekeeping gene promoters. CpG islands associated with housekeeping genes generally remain unmethylated throughout development and are constitutively enriched with active chromatin marks, thereby permitting continuous transcriptional activity across diverse cell types. The A2UCOE element, derived from the bidirectional promoter shared by HNRPA2B1 and CBX3, retains these structural characteristics, including high CpG density, constitutive chromatin accessibility, and resistance to DNA methylation [11,25,29]. Preservation of these genomic features is thought to facilitate the maintenance of an open chromatin configuration and protect adjacent promoters from epigenetic silencing following vector integration [19].

Another possible explanation for the performance of the minimal 0.5 kb construct is that the essential functional motifs required for chromatin opening are concentrated within a relatively small genomic region rather than being uniformly distributed across the entire classical UCOE sequence. Previous deletion and functional analyses have suggested that not all regions of the original A2UCOE contribute equally to anti-silencing activity and that shorter derivatives may retain substantial biological function when critical CpG-rich domains are preserved [19,24]. The ability of the 0.5 kb fragment to maintain stable transgene expression throughout both the pluripotent and neuronal stages observed in the present study supports this hypothesis and suggests that the functional boundaries of UCOEs may be further refined without compromising their anti-silencing capacity.

An additional consideration is the profound epigenetic remodeling that accompanies neuronal differentiation. During lineage commitment, pluripotent stem cells undergo extensive changes in DNA methylation, nucleosome positioning, chromatin accessibility, and histone modification patterns, ultimately leading to activation of neuronal gene networks while silencing pluripotency-associated loci [8,21]. Such dynamic chromatin reorganization frequently compromises expression from integrated viral vectors [13,21]. Nevertheless, persistent eGFP expression was maintained throughout neuronal differentiation in the present study, indicating that the protective effect of UCOEs extends beyond the undifferentiated state and remains effective during developmental epigenetic remodeling. This observation is particularly relevant for regenerative medicine and stem cell-based gene therapy applications, where durable transgene expression following terminal differentiation is essential for long-term therapeutic efficacy [2,17].

From a vector-engineering perspective, the present findings highlight the potential utility of a compact UCOE that preserves long-term transgene expression while minimizing the genetic footprint within the lentiviral vector. Packaging capacity remains a major limitation for viral vector-based gene therapy; although lentiviral vectors can accommodate larger inserts than adeno-associated viral (AAV) vectors, the incorporation of multiple regulatory elements, therapeutic transgenes, and safety switches rapidly approaches the upper packaging limit [4,10,25]. The 0.5 kb UCOE retains substantial anti-silencing activity while occupying less than one-third of the sequence required for conventional UCOE constructs, which could be further evaluated for applications requiring additional vector capacity for therapeutic or regulatory components. This feature is particularly attractive for next-generation lentiviral vectors designed for complex therapeutic applications requiring multiple genetic elements within a single construct [7,19,24].

Stable transgene expression throughout differentiation is especially critical in stem cell-based gene therapy, where transplanted cells must maintain therapeutic gene expression for prolonged periods. Human iPSCs undergo extensive proliferation and differentiation before reaching their terminal phenotype, during which integrated transgenes frequently become susceptible to progressive epigenetic silencing [2,3,18]. The preservation of reporter expression throughout neuronal differentiation and maturation provides a rationale for further evaluating compact UCOE constructs in neuronal cell-based models relevant to neurological disorders. where cell replacement strategies based on iPSC-derived neurons are under active investigation [8,16]. Furthermore, the reduced size of the 0.5 kb element may also warrant further evaluation in vector configurations incorporating additional regulatory or genome-engineering components that increasingly compete for lentiviral vector space. Collectively, the present findings indicate that functional UCOE minimization offers advantages beyond simple vector size reduction, supporting the development of more compact, versatile, and potentially translatable gene delivery systems for regenerative medicine and neurological gene therapy [7,12].

Although the present study demonstrates the potential of a minimized UCOE sequence to preserve long-term transgene expression in human induced pluripotent stem cells and throughout neuronal differentiation, several limitations should be acknowledged.

First, transgene expression was evaluated primarily through eGFP reporter activity, mean fluorescence intensity, and vector copy number analyses. While these approaches provide robust and widely accepted measures of transgene stability, they do not directly characterize the epigenetic mechanisms responsible for transcriptional maintenance. Previous studies have demonstrated that UCOEs preserve transgene expression by resisting DNA methylation and maintaining transcriptionally permissive chromatin states; however, these mechanisms were not directly investigated in the present work [3,6,19]. Future studies incorporating genome-wide DNA methylation profiling, bisulfite sequencing, chromatin immunoprecipitation sequencing (ChIP-seq) for active and repressive histone modifications, and chromatin accessibility assays such as ATAC-seq would provide important mechanistic insight into how the minimized UCOE preserves long-term transcriptional competence.

A further limitation of the present study is the relatively modest number of independent biological replicates used for some experiments. A formal prospective power calculation was not performed because the present study was designed as an exploratory vector-characterization and proof-of-concept investigation. The number of biological replicates was selected based on the reproducibility of independent transduction and differentiation experiments and the practical feasibility of maintaining longitudinal human iPSC cultures over extended experimental periods. Although individual biological replicate datapoints are presented to demonstrate biological variability, the modest sample size limits statistical power for detecting small differences among constructs. Therefore, the comparative findings should be interpreted as evidence of comparable performance under the experimental conditions tested rather than as evidence of formal statistical equivalence.

Second, functional neuronal maturation was not directly assessed by electrophysiological recordings, calcium imaging, neurotransmitter release measurements, or synaptic activity analyses. Although the observed neuronal morphology and expression of TUJ1, TH, and GIRK2 provide phenotypic characterization of neuronal subpopulations, these measurements do not establish electrophysiological maturity or functional synaptic activity. Accordingly, the present findings demonstrate persistence of reporter expression during neuronal differentiation and morphological maturation but do not establish functional neuronal maturity. Dedicated electrophysiological and calcium-imaging studies will be required in future work to determine whether sustained transgene expression is maintained in functionally mature neuronal populations. In addition, neuronal differentiation efficiency was not quantitatively compared between eGFP-positive and eGFP-negative populations; therefore, the present study cannot determine whether vector transduction or UCOE incorporation influences neuronal differentiation efficiency.

Related to this, the neuronal cultures generated here were produced using a general dual-SMAD-based induction protocol that did not include ventral midbrain patterning cues such as sonic hedgehog agonists, FGF8, or GSK3β inhibition. Although TH-positive cells, a subset of which co-expressed GIRK2, were present in the differentiated cultures, these findings do not establish a fully specified ventral midbrain dopaminergic identity because the differentiation protocol did not include directed ventral midbrain patterning cues or additional midbrain-associated markers such as FOXA2, LMX1A, or NURR1. The neuronal characterisation presented here should therefore be regarded as descriptive evidence that transgene expression persists through neuronal commitment and maturation, rather than as a demonstration that the constructs sustain expression in a defined midbrain dopaminergic population. Evaluation of the minimal UCOE in a dedicated, patterned dopaminergic differentiation protocol would be a valuable extension of this work, particularly for gene therapy applications targeting Parkinson’s disease [8].

Importantly, quantitative analysis demonstrated that TH and GIRK2 expression was restricted to subpopulations of the differentiated neuronal cultures rather than being uniformly present. Approximately 23.4% of TUJ1-positive neurons exhibited TH immunoreactivity, and 31.8% of TH-positive cells co-expressed GIRK2. These findings are consistent with the heterogeneous nature of the cultures generated using the general neuronal differentiation protocol employed here. Since ventral midbrain patterning factors were not incorporated into the differentiation procedure, the presence of TH- and GIRK2-positive cells should not be interpreted as evidence of directed dopaminergic specification. Rather, these markers provide phenotypic characterization of neuronal subpopulations arising within the differentiated cultures.

Another limitation is that the present investigation utilized a single human iPSC line and one lentiviral vector backbone. Although this experimental design minimized biological variability and enabled direct comparison among different UCOE constructs, validation in multiple independent pluripotent stem cell lines, primary human cells, and alternative viral vector systems would be necessary to establish the broader applicability and reproducibility of the observed effects [2,18,20]. Likewise, evaluation of the minimized UCOE in additional differentiated cell types, including astrocytes, oligodendrocytes, cardiomyocytes, and hematopoietic cells, would further clarify its versatility across diverse therapeutic applications and facilitate its future translation into clinically relevant gene therapy platforms [11,17,24].

The duration of the present study was limited to up to 60 days of culture before and during neuronal differentiation. Although this period is sufficient to demonstrate early and intermediate transgene stability, gene therapy applications frequently require therapeutic gene expression that remains stable for months or even years. Therefore, long-term in vitro culture together with in vivo transplantation studies will be necessary to determine whether the anti-silencing properties of the minimized UCOE remain effective over clinically relevant time scales [2,7,17]. Such studies will also be important for evaluating the long-term safety, stability, and therapeutic durability of UCOE-containing lentiviral vectors under physiological conditions [4,25].

Although the present findings demonstrate sustained UCOE-associated transgene expression during prolonged in vitro culture and neuronal differentiation, translation of this effect to in vivo settings represents an important additional challenge. The chromatin environment encountered by an integrated expression cassette may vary substantially among tissues, cell types, developmental states, and disease conditions, potentially influencing the capacity of a UCOE to maintain an accessible transcriptional state. In addition, vector biodistribution, transduction efficiency, cellular turnover, immune responses to vector- or transgene-associated components, and selection of the target cell population may influence the magnitude and durability of transgene expression in vivo. For integrating lentiviral vectors, long-term genomic safety, including integration-site distribution and potential insertional effects, also requires careful evaluation. Future studies should therefore assess the UCOE constructs in appropriate preclinical models using longitudinal measurements of transgene expression and vector persistence together with tissue-specific, immunological, and genomic safety analyses. Optimization of the UCOE–promoter–transgene configuration, vector dose, delivery strategy, and target-cell specificity may further facilitate translation. Consequently, the current results should be regarded as in vitro proof-of-concept evidence supporting the potential of these UCOEs to promote sustained transgene expression, while their long-term efficacy and safety in vivo remain to be established.

Finally, although the present study demonstrates that the 0.5 kb construct performs comparably to the larger UCOE fragments under the experimental conditions employed, the precise sequence determinants responsible for its biological activity remain to be identified. Systematic deletion mapping, mutational analyses of CpG-rich regions, and computational identification of transcription factor-binding sites may facilitate the definition of the minimal functional domain required for chromatin-opening activity. Such information would not only improve our understanding of UCOE biology but also support the rational design of next-generation synthetic chromatin-opening elements optimized for clinical gene therapy applications [7,19,24].

Despite these limitations, the present work provides experimental evidence supporting the feasibility of substantial UCOE sequence minimization while preserving long-term transgene expression in human induced pluripotent stem cells and throughout neuronal differentiation. Collectively, these findings provide in vitro proof-of-concept evidence that substantial UCOE sequence minimization can preserve long-term transgene expression in human iPSCs and during neuronal differentiation. The 0.5 kb UCOE therefore represents a candidate compact regulatory element for further evaluation in lentiviral vector engineering. Additional studies in multiple cell types, extended culture and in vivo models will be required to determine its broader applicability, functional performance, and long-term safety [2].

## 4. Materials and Methods

### 4.1. Construction and Validation of UCOE Lentiviral Vectors/Sanger Sequencing Validation of the 0.5-kb UCOE Construct

Chemically competent *Escherichia coli* DH5α cells (Life Technologies Ltd., Carlsbad, CA, USA) were used for plasmid propagation and cloning procedures. All bacterial manipulations were performed under aseptic conditions. Transformed bacteria were cultured on Luria–Bertani (LB) agar plates supplemented with 100 μg/mL ampicillin and incubated overnight at 37 °C. Single colonies were subsequently expanded in LB broth containing the same concentration of ampicillin with continuous shaking (180–200 rpm) at 37 °C. Agar plates and liquid cultures were maintained at 4 °C for short-term storage (up to two weeks), whereas long-term bacterial stocks were preserved at −80 °C in LB medium supplemented with 20% glycerol [30].

Three next-generation UCOE constructs containing 1.7 kb, 1.2 kb, and 0.5 kb chromatin-opening fragments were generated using the same approximately 8 kb plasmid backbone, with the inserted UCOE fragment representing the only difference among constructs. Plasmid DNA was isolated from overnight bacterial cultures using QIAGEN MiniPrep or MaxiPrep kits (QIAGEN, Hilden, Germany) according to the manufacturer’s instructions. DNA concentration and purity were determined spectrophotometrically using a NanoDrop instrument (Thermo Fisher Scientific, Wilmington, DE, USA), and purified plasmids were stored at −20 °C until further use [31].

The 0.5 kb, 1.2 kb, and 1.7 kb UCOE fragments were commercially synthesized and inserted into the plasmid backbone without PCR amplification. Successful cloning was assessed by analytical restriction enzyme digestion using SfiI, EcoRV, ClaI, BamHI, and AgeI, followed by agarose gel electrophoresis. Clones exhibiting restriction patterns consistent with the predicted plasmid maps were selected for subsequent lentiviral vector production.

Restriction digestion products were separated on 0.8–2% agarose gels prepared in 1× TAE buffer containing 0.005% ethidium bromide. Electrophoresis was performed under standard conditions, and DNA fragments were visualized under ultraviolet illumination. Fragment sizes were estimated using 100 bp and 1 kb DNA ladders (New England Biolabs, Ipswich, MA, USA). Restriction reactions contained 1–40 μg plasmid DNA, the appropriate NEB reaction buffer, bovine serum albumin when required, and restriction enzymes at a ratio of 1 U per μg DNA, with enzyme volume maintained below 10% of the total reaction.

Sanger sequencing validation of the 0.5-kb UCOE construct. Because the newly developed 0.5-kb CBX3-derived UCOE represents the principal novel regulatory element evaluated in this study, nucleotide-level sequence verification was performed on the final recombinant lentiviral transfer plasmid carrying the 0.5-kb UCOE construct. Six overlapping sequencing amplicons were designed to cover the vector–0.5UCOE junction (432 bp), the internal 0.5-kb UCOE region (512 bp forward and 509 bp reverse), the 0.5UCOE–PGK promoter junction (478 bp), the PGK–eGFP junction (556 bp), and the eGFP–WPRE junction (501 bp). The sequencing primers were Vector-F (5′-GACCTGAAAGCGGCCGCGGC-3′), UCOE-F (5′-GCGGCCGCCGGGCGGCGGCT-3′), UCOE-R (5′-GGCCGCGGGCGCCCGCGGCG-3′), PGK-F (5′-GGGTAGGGGAGGCGCTGCGG-3′), eGFP-R (5′-CTCGCCCTTGCTCACCATGG-3′), and WPRE-R (5′-CTTCCATAGAGCCCACCGCA-3′). The resulting Sanger sequencing reads were aligned with the corresponding reference construct sequence to assess nucleotide-level sequence integrity and to identify potential nucleotide substitutions, insertions, or deletions. Representative sequence alignment summaries and electropherogram traces are provided in Supplementary Figure S1. Because the complete nucleotide sequence of the newly developed UCOE fragments is subject to institutional intellectual property protection, full sequence coordinates are not disclosed in the manuscript. This restriction applies only to public disclosure of the proprietary sequence information and does not affect nucleotide-level verification of the construct.

### 4.2. Lentiviral Vector Production and Viral Titration

All lentiviral vectors were generated using a third-generation self-inactivating (SIN) lentiviral system containing a deletion within the U3 region of the 3′ long terminal repeat (δU3) to enhance biosafety and eliminate promoter activity following genomic integration. All UCOE fragments were derived from the human HNRPA2B1–CBX3 locus. Each vector contained one of the three UCOE fragments (1.7 kb, spanning the HNRPA2B1–CBX3 dual-promoter region; 1.2 kb, a truncated derivative of the same region; or 0.5 kb, a minimal promoterless CpG-rich fragment derived from the CBX3 side of the locus) positioned upstream of an internal phosphoglycerate kinase (PGK) promoter driving expression of the enhanced green fluorescent protein (eGFP) reporter gene [32]. All constructs analysed in this study used eGFP as the reporter, so that fluorescence data were directly comparable across groups. Two reference vectors were included throughout: a UCOE-less vector carrying only the PGK–eGFP cassette (negative control) and the previously characterised A2UCOE-SEW vector, which contains the full-length 2.2 kb A2UCOE upstream of an SFFV–eGFP–WPRE cassette and served as the positive anti-silencing control.

Human embryonic kidney 293T (HEK293T) cells were maintained in Dulbecco’s Modified Eagle Medium (DMEM) supplemented with 10% fetal calf serum (FCS), 2 mM L-glutamine, and 1% penicillin–streptomycin (Life Technologies Ltd.) at 37 °C in a humidified atmosphere containing 5% CO_2_. Cells were seeded into T162 culture flasks (Corning, Ewloe, UK) at a density of 2 × 10^7^ cells per flask and transfected at approximately 80–90% confluence.

For each flask, plasmid DNA containing the lentiviral transfer vector, envelope plasmid (pVSV-G; 50 μg), and packaging plasmid (p8.74; 32.5 μg) was diluted in Opti-MEM (Life Technologies Ltd.). Polyethylenimine (PEI) was prepared separately in Opti-MEM, sterile-filtered through a 0.2 μm membrane, combined with the DNA solution at a 1:1 ratio, and incubated for 20 min at room temperature to allow complex formation. HEK293T cells were washed with Opti-MEM prior to transfection, after which the DNA–PEI complexes were added. Four hours later, the transfection medium was replaced with complete DMEM, and fresh medium was supplied again after 24 h [32].

Lentiviral supernatants were harvested at 48 and 72 h post-transfection, filtered through 0.2 μm membrane filters, and concentrated by ultracentrifugation at 25,000× *g* for 2 h at 4 °C. Viral pellets were gently resuspended in Opti-MEM, aliquoted, and stored at −80 °C until use.

Functional viral titers were determined by flow cytometry. Briefly, HEK293T cells were seeded into 24-well plates and transduced with serial 10-fold dilutions of concentrated viral stocks in the presence of polybrene (final concentration, 8 μg/mL). Untransduced cells served as negative controls. Seventy-two hours after transduction, the percentage of eGFP-positive cells was determined by flow cytometry, and viral titers were calculated as transducing units per milliliter (TU/mL). Dilutions producing 1–20% eGFP-positive cells were used for titer calculations to minimize the probability of multiple vector integration events per cell and ensure accurate estimation of infectious viral particles [17,32].

### 4.3. Flow Cytometric Analysis

For viral titration, adherent HEK293T cells were washed with phosphate-buffered saline (PBS) and dissociated using TrypLE™ Express (Life Technologies Ltd.). Cell suspensions were neutralized with serum-containing medium, collected into centrifuge tubes, and pelleted at 1000 rpm for 5 min. Cell pellets were resuspended in PBS containing 4% formaldehyde and fixed at 4 °C in the dark until analysis.

A minimum of 2 × 10^5^ cells per sample was analyzed using a BD FACSVerse flow cytometer (BD Biosciences, Franklin Lakes, NJ, USA). Cell populations were initially identified based on forward scatter (FSC) and side scatter (SSC) characteristics to exclude debris and cell aggregates. eGFP fluorescence was detected in the FL1 channel (525 nm), whereas FL2 (575 nm) was used to exclude spectral overlap where appropriate. Viral titers were calculated from samples containing 1–20% eGFP-positive cells, thereby minimizing the probability of multiple vector integration events and allowing accurate determination of infectious viral particles. Data were analyzed using FlowJo software, version 10.8.1 (FlowJo LLC, Ashland, OR, USA) [29].

Flow-cytometric analysis of reporter expression. The percentage of eGFP-positive cells and eGFP mean fluorescence intensity (MFI) were evaluated as separate flow-cytometric parameters. The percentage of eGFP-positive cells represents the proportion of cells exceeding the predefined eGFP positivity threshold, whereas MFI represents the mean fluorescence intensity of the eGFP-positive population. Raw MFI values obtained from the flow-cytometry analysis software were reported in arbitrary fluorescence units (a.u.) without percentage transformation or normalization to baseline values. A representative gating strategy and representative primary flow-cytometry plots are provided in Supplementary Figure S2.

To characterize the pluripotent phenotype, iPSCs were dissociated into single-cell suspensions using Gentle Cell Dissociation Reagent (STEMCELL Technologies, Vancouver, BC, Canada), fixed and permeabilized using Fix/Perm buffer, and incubated for 50 min at 4 °C in the dark. Cells were subsequently washed with Perm/Wash buffer and stained on ice for 50 min with antibodies against SOX2, OCT3/4, SSEA4, and TRA-1-60 together with the corresponding isotype controls. Flow cytometric data were acquired using a BD FACSVerse instrument (BD Biosciences) and analyzed with FlowJo software.

### 4.4. Human iPSC Culture and Characterization

Human induced pluripotent stem cells (ATCC ACS-1030) were obtained from the American Type Culture Collection (ATCC, Manassas, VA, USA). Upon receipt, cells were expanded according to the manufacturer’s recommendations and cryopreserved in liquid nitrogen until use.

For all experiments, iPSCs were cultured under feeder-free conditions on Matrigel-coated six-well plates (Corning, Ewloe, UK, Cat. No. 354277). Matrigel was diluted 1:100 in accordance with the manufacturer’s instructions and incubated for 1 h at 37 °C before cell seeding. Cells were maintained in mTeSR™1 medium (STEMCELL Technologies, Cat. No. 05850), with daily medium replacement, at 37 °C in a humidified incubator containing 5% CO_2_.

Cells were passaged every 4–5 days or upon reaching approximately 80–90% confluence using a non-enzymatic EDTA-based dissociation protocol. Following passage, cultures were maintained in medium supplemented with 10 μM ROCK inhibitor Y-27632 (Selleckchem, Houston, TX, USA) for the first 24 h to improve cell survival. Cell morphology and colony integrity were monitored daily using phase-contrast microscopy, and spontaneously differentiated areas were manually removed when necessary.

For comparative characterization of the effects of lentiviral transduction, a parallel population of untransduced human iPSCs was maintained as a negative control. Untransduced and transduced cultures were maintained under the same feeder-free culture conditions and processed in parallel for morphological, immunofluorescence, and molecular analyses. The pluripotent phenotype was further confirmed by immunofluorescence staining. Cells cultured on chamber slides were fixed in 4% paraformaldehyde, permeabilised with 0.1% Triton X-100, blocked in normal goat serum, and incubated overnight at 4 °C with primary antibodies against TRA-1-60 (mouse monoclonal, Millipore, Burlington, MA, USA, Cat. No. MAB4360, 1:200), SOX2 (rabbit monoclonal, Cell Signaling Technology, Danvers, MA, USA, Cat. No. 3579, 1:400), and NANOG (rabbit monoclonal, Cell Signaling Technology, Cat. No. 4903, 1:400). Alexa Fluor 594-conjugated goat anti-mouse IgG (Invitrogen, Waltham, MA, USA, Cat. No. A-11005, 1:500) and Alexa Fluor 647-conjugated goat anti-rabbit IgG (Invitrogen, Cat. No. A-21245, 1:500) were used as secondary antibodies (1 h, room temperature). Primary-antibody-omitted preparations served as negative controls. Cell nuclei were counterstained with DAPI, and images were acquired on a Zeiss LSM 710 (Carl Zeiss, Oberkochen, Germany) laser scanning confocal microscope using identical acquisition settings for all groups within an experiment [33]. TRA-1-60, SOX2, and NANOG immunostainings were performed on separate coverslips to avoid species-related limitations in multiplex immunofluorescence. Representative images from independent stainings are shown in Figure 5. For quantitative fluorescence analysis, images from untransduced and transduced cultures were acquired using identical laser power, detector gain, pinhole, magnification, and other acquisition parameters within each experiment. Fluorescence intensity was quantified from comparable fields using the same image-processing and analysis settings for all groups. Background fluorescence was subtracted before measurement, and the resulting fluorescence intensity values were used for comparison of TRA-1-60, SOX2, and NANOG expression between untransduced and transduced cultures. Three independent biological replicates were analyzed (*n* = 3).

Untransduced iPSCs maintained under the same culture conditions were included as a negative control for lentiviral reporter expression and as a reference group for pluripotency-marker analysis. For quantitative fluorescence analysis, images from untransduced and transduced cultures were acquired using identical microscope settings within each experiment. Fluorescence intensity was quantified using the same image-processing and analysis parameters for all groups. Independent biological replicates were analyzed, and the mean fluorescence intensity was used for statistical comparison between groups.

### 4.5. Lentiviral Transduction of Human iPSCs

Human iPSCs were transduced with lentiviral vectors carrying the different UCOE constructs. Functional viral titers determined in HEK293T cells were used to calculate the multiplicity of infection (MOI). Approximately 1 × 10^4^ viable cells were seeded per well in 24-well plates and transduced after cell attachment using MOIs ranging from 1 to 5. Following a 4-h incubation period, the transduction medium was replaced with fresh iPSC culture medium, which was renewed again after 24 h.

Transduction efficiency was initially evaluated by fluorescence microscopy, and vector doses were optimized to achieve the desired percentage of eGFP-positive cells while minimizing multiple vector integration events. When required, additional batches of lentiviral vectors were generated using identical production conditions.

Following transduction, iPSCs were monitored by fluorescence microscopy and confocal microscopy to evaluate reporter gene expression and maintenance of typical pluripotent colony morphology. Cells were subsequently harvested for flow cytometric analysis and downstream vector copy number determination. Experimental conditions were maintained consistently throughout the study to minimize variability associated with lentiviral integration and transgene expression [17,34].

### 4.6. Maintenance of Transduced iPSCs and Longitudinal Expression Analysis

Following lentiviral transduction and determination of the initial percentage of eGFP-positive cells, transduced iPSC cultures were maintained under feeder-free conditions in mTeSR™1 medium supplemented with the appropriate pluripotency maintenance factors according to the experimental design. Cells were cultured under standard conditions for 45 days, with routine medium replacement and passaging as required. Throughout this manuscript, the 45-day period refers to the undifferentiated (pluripotency-maintenance) arm of the study, in which transduced iPSCs were kept in mTeSR™1 without differentiation. A parallel arm of transduced cells was committed to neuronal differentiation on Day 5 post-transduction and followed to Day 60; results from that arm are reported in Section 2.4 and Section 2.5. The two arms therefore share the same transduction event and the same Day 3 baseline but differ in culture fate thereafter.

For longitudinal analysis, independent cultures were established for each lentiviral construct. At weekly intervals, cells were harvested for flow cytometric quantification of eGFP expression and genomic DNA isolation for vector copy number (VCN) analysis. Samples were collected on the same day each week throughout the 45-day observation period to ensure consistency between experimental time points.

**Figure 15:**
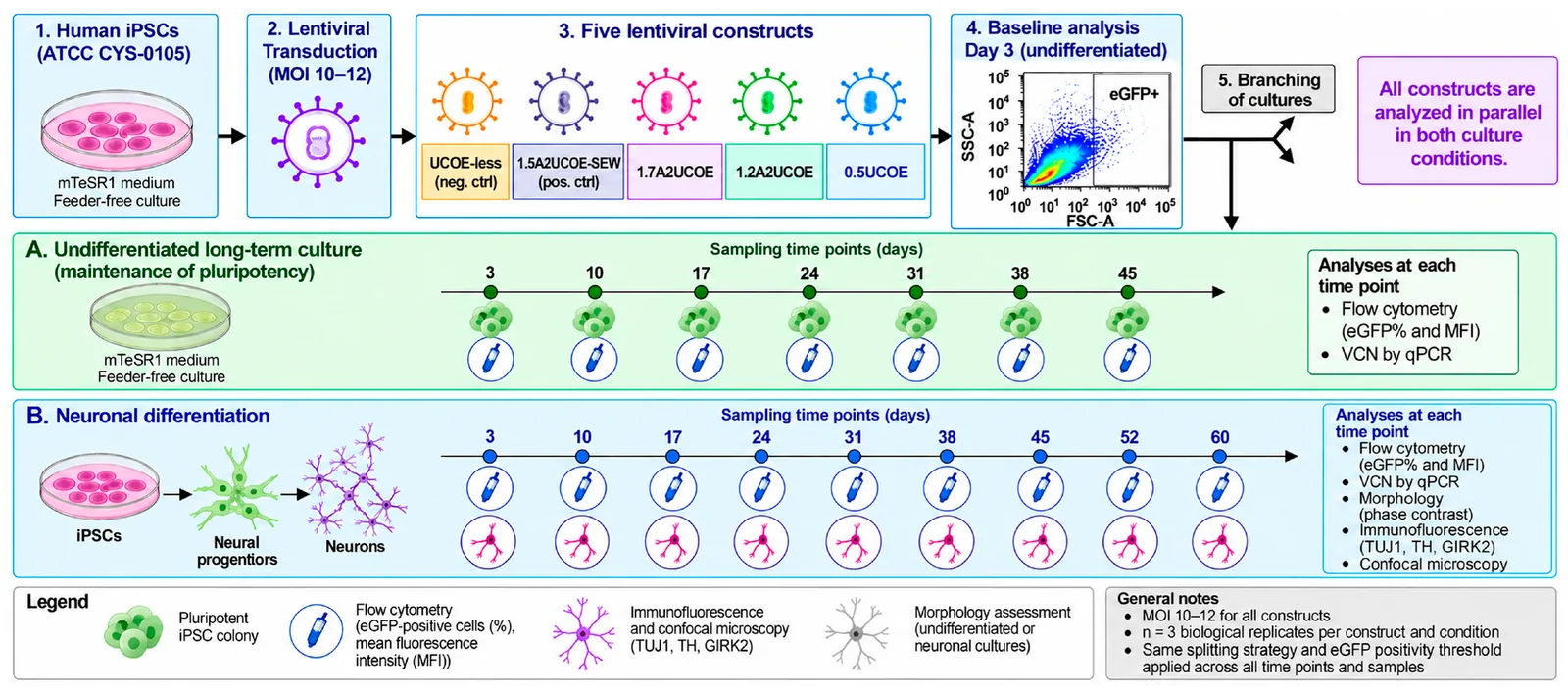
Schematic overview of the experimental workflow and longitudinal culture timeline. Human iPSCs were initially transduced with the UCOE-less, 1.5A2UCOE-SEW, 1.7A2UCOE, 1.2A2UCOE, or 0.5UCOE lentiviral constructs. Following transduction, cultures were evaluated at the Day 3 baseline and subsequently branched into two experimental arms. In the undifferentiated arm, transduced iPSCs were maintained under pluripotency conditions for 45 days, with longitudinal assessment of eGFP-positive cell percentage, eGFP mean fluorescence intensity (MFI), and vector copy number (VCN). In the neuronal differentiation arm, neuronal differentiation was initiated on Day 5 post-transduction and cultures were followed through Day 60, including neural induction, neural precursor cell expansion, and terminal neuronal differentiation. Longitudinal eGFP expression and VCN were assessed throughout this arm, while morphological and immunofluorescence/confocal analyses were performed during neuronal differentiation and maturation. The schematic indicates the major experimental time points, culture branching, sampling points, and corresponding downstream analyses.

The overall experimental design and longitudinal workflow are summarized schematically in Figure 15.

### 4.7. Neuronal Differentiation and Neural Precursor Cell Induction

To evaluate transgene stability during lineage commitment, transduced human iPSCs were differentiated into neural cells using a commercially available neuronal differentiation protocol. Following optimization of transduction efficiency, neuronal differentiation was initiated on Day 5 after transduction.

Cells were cultured on Matrigel-coated plates under feeder-free conditions until reaching approximately 80–90% confluence. Neural induction was performed using a defined neural induction medium consisting of DMEM/F12 supplemented with N2 supplement, L-glutamine, non-essential amino acids, and heparin (2 μg/mL). Dual-SMAD inhibition was achieved by supplementing the medium with SB431542 (10 μM) and LDN-193189 (100 nM). Culture medium was replaced daily throughout the induction period.

After seven days of neural induction, neural rosettes were manually isolated, dissociated using Accutase, and replated in Neural Precursor Cell (NPC) medium. NPC cultures were expanded for an additional 7–10 days before terminal neuronal differentiation.

For descriptive purposes, sequential stages of early neuronal differentiation were operationally designated according to the timing of the differentiation protocol and the predominant cellular morphology observed at each time point. Day 2 was designated as the neural induction stage based on the initial loss of compact iPSC colony morphology following exposure to neural induction conditions. Day 4 was designated as the early NPC stage based on the emergence of neural progenitor-like morphological features, without implying definitive molecular confirmation of NPC identity. Day 6 was designated as the NPC expansion stage based on the increased prevalence and spatial expansion of cells exhibiting progenitor-like morphology. Day 8 was designated as the early neuronal stage based on the emergence of elongated neuronal-like cells and neuritic extensions, together with detectable βIII-tubulin (TUJ1) immunoreactivity. These designations represent operational stages of the in vitro differentiation protocol and do not imply homogeneous or completely stage-restricted cell populations.

### 4.8. Differentiation and Characterization of Mature Neurons

Neural precursor cells were differentiated into mature neurons by replacing the expansion medium with neuronal differentiation medium containing Neurobasal medium supplemented with B27, GlutaMAX, brain-derived neurotrophic factor (BDNF), glial cell-derived neurotrophic factor (GDNF), cyclic AMP (cAMP), ascorbic acid, and DAPT. Basic fibroblast growth factor (bFGF) and epidermal growth factor (EGF) were omitted during terminal differentiation. Culture medium was replaced every other day, and neuronal maturation was continued for 14–21 days.

Neuronal differentiation and neuronal subtype identity were assessed by immunofluorescence staining for the mature neuronal marker βIII-tubulin (TUJ1) and for the dopaminergic markers tyrosine hydroxylase (TH) and G protein-activated inward rectifier potassium channel 2 (GIRK2).

For immunocytochemistry, differentiated cultures grown on Matrigel-coated coverslips or chamber slides were fixed in 4% paraformaldehyde for 15 min at room temperature, washed in PBS, permeabilised with 0.1% Triton X-100 for 10 min, and blocked for 1 h at room temperature in PBS containing normal goat serum and bovine serum albumin. Preparations were then incubated overnight at 4 °C with primary antibodies against βIII-tubulin (TUJ1) (mouse monoclonal, BioLegend, San Diego, CA, USA, Cat. No. 801202, 1:500), tyrosine hydroxylase (TH) (rabbit polyclonal, Millipore, Cat. No. AB152, 1:500), and GIRK2 (guinea pig polyclonal, Alomone Labs, Jerusalem, Israel, Cat. No. APC-006-GP, 1:200).

After washing, cells were incubated for 1 h at room temperature with species-specific Alexa Fluor-conjugated secondary antibodies. βIII-tubulin (TUJ1) was detected with Alexa Fluor 594-conjugated goat anti-mouse IgG, tyrosine hydroxylase (TH) with Alexa Fluor 647-conjugated goat anti-rabbit IgG, and GIRK2 (guinea pig) with Alexa Fluor 555-conjugated goat anti-guinea pig IgG. Because eGFP occupied the green channel, all immunostainings were acquired in spectrally distinct red or far-red channels. Nuclei were counterstained with DAPI, and preparations in which the primary antibody was omitted served as negative controls.

Images were acquired on a Zeiss LSM 710 (Carl Zeiss, Oberkochen, Germany) laser scanning confocal microscope with identical laser power, gain, and pinhole settings across all experimental groups within a given experiment. Representative co-expression of eGFP with βIII-tubulin and TH was evaluated on single confocal optical sections rather than on maximum-intensity projections, and representative fields were selected from at least three independent differentiations.

Quantitative analysis of neuronal marker expression was performed using confocal images obtained from three independent biological replicates (*n* = 3). Multiple non-overlapping microscopic fields were analyzed for each biological replicate using identical image-acquisition settings within each staining experiment. Thresholds for marker positivity were established using the corresponding negative controls and were applied consistently to images within the same experimental series. Nuclei were identified by DAPI staining to assist with cell identification.

TUJ1-positive neuronal cells and TH-positive cells were identified from their respective fluorescence channels. The proportion of TH^+^/TUJ1^+^ cells was calculated as the number of TH-positive cells among the TUJ1-positive neuronal population × 100. The proportion of GIRK2^+^/TH^+^ cells was calculated as the number of GIRK2-positive cells among the TH-positive population × 100. Measurements obtained from individual microscopic fields were summarized for each independent biological replicate, and biological replicate values were used for statistical presentation. Data are presented as mean ± SEM.

### 4.9. Vector Copy Number Analysis

Genomic DNA was isolated from transduced human iPSCs and used for quantitative PCR analysis of lentiviral vector copy number (VCN), as previously described [17]. A previously characterized single-copy eGFP-positive clonal cell population was used as the reference standard for calibration. Quantitative PCR was performed using WPRE as the vector-specific target and the endogenous human albumin (ALB) gene as the genomic reference, with primers WPRE-F (5′-GTCCTTTCCATGGCTGCTC-3′) and WPRE-R (5′-CCGAAGGGACGTAGCAGA-3′) for the target, and ALB-F (5′-TTTGCAGATGTCAGTGAAAGAGA-3′) and ALB-R (5′-TGGGGAGGCTATAGAAAATAAGG-3′ for the reference. Standard curves were generated using serially diluted reference DNA samples, and VCN was calculated based on the relationship between the lentiviral target and the genomic reference. qPCR reactions were performed using PowerUp SYBR Green Master Mix (Thermo Fisher Scientific, Waltham, MA, USA, Cat. No. A25742) on an Applied Biosystems QuantStudio 5 Real-Time PCR System (Applied Biosystems, Foster City, CA, USA). Each reaction was performed in a final volume of 20 µL containing 10 µL of 2× PowerUp SYBR Green Master Mix, forward and reverse primers at a final concentration of 1 µM each, 100 ng of genomic DNA, and nuclease-free water. The amplification program consisted of an initial denaturation at 95 °C for 5 min, followed by 45 cycles of denaturation at 95 °C for 10 s, annealing at 60 °C for 10 s, and extension at 72 °C for 10 s. Each sample was analyzed in technical triplicate.

### 4.10. RT-qPCR Analysis of Pluripotency-Associated Gene Expression

RNA was isolated from untransduced and transduced human iPSCs using the RNeasy Mini Kit (QIAGEN, Hilden, Germany). RNA concentration and purity were assessed using a NanoDrop 2000 spectrophotometer (Thermo Fisher Scientific, Waltham, MA, USA). For reverse transcription, the High-Capacity cDNA Reverse Transcription Kit (Applied Biosystems, Thermo Fisher Scientific) was used according to the manufacturer’s instructions, using 1 µg of total RNA per reaction.

Quantitative real-time PCR was performed using PowerUp SYBR Green Master Mix (Thermo Fisher Scientific, Cat. No. A25742) on an Applied Biosystems QuantStudio 5 Real-Time PCR System. Gene expression analysis was performed for POU5F1, SOX2, and NANOG, with GAPDH used as the reference gene. The following primer sequences were used: POU5F1-F, 5′-ACCCACACTGCAGCAGATCA-3′ and POU5F1-R, 5′-CCACACTCGGACCACATCC-3′; SOX2-F, 5′-GGGGGAATGGACCTTGTATAG-3′ and SOX2-R, 5′-GCAAAGCTCCTACCGTACCA-3′; NANOG-F, 5′-ACAACTGGCCGAAGAATAGCA-3′ and NANOG-R, 5′-GGTTCCCAGTCGGGTTCAC-3′. GAPDH was amplified using GAPDH-F, 5′-GAAGGTGAAGGTCGGAGTC-3′ and GAPDH-R, 5′-GAAGATGGTGATGGGATTTC-3′, with each primer at a final concentration of 300 nM. Relative transcript expression was calculated using the 2^−ΔΔCt^ method. Each biological sample was analyzed using technical triplicate. Thermal cycling consisted of UDG activation at 50 °C for 2 min, followed by Dual-Lock DNA polymerase activation at 95 °C for 2 min and 40 cycles of denaturation at 95 °C for 3 s and annealing/extension at 60 °C for 30 s. Three independent biological replicates were analyzed.

### 4.11. Statistical Analysis

Statistical analyses were performed using GraphPad Prism version 7.0 (GraphPad Software, San Diego, CA, USA). Quantitative experiments were performed using three to four independent biological replicates (*n* = 3–4), with the exact number indicated in the corresponding figure legends. Data are presented as mean ± standard error of the mean (SEM), with individual biological replicate datapoints shown where applicable. Normality of data distribution was assessed using the Shapiro–Wilk test prior to parametric analyses.

For longitudinal experiments in which the same biological replicates were followed across multiple time points, three independent biological replicates (*n* = 3) were used, with each replicate representing an independent transduction and differentiation experiment. A formal prospective power calculation was not performed because the present study was designed as an exploratory vector-characterization and proof-of-concept study. The number of biological replicates was selected based on the reproducibility of independent transduction and differentiation experiments and the practical feasibility of maintaining longitudinal human iPSC cultures over extended experimental periods. Individual biological replicate datapoints were presented to provide transparency regarding biological variability. For these longitudinal experiments, two-way repeated-measures analysis of variance (ANOVA) was used, with experimental construct and time as the two factors. The construct × time interaction was evaluated to determine whether temporal changes differed among constructs. When significant main effects or interactions were detected, Tukey’s multiple-comparisons test was used for post hoc pairwise comparisons.

For comparisons involving multiple independent groups without repeated longitudinal measurements, one-way ANOVA followed by Tukey’s multiple-comparisons test was used, as appropriate. Comparisons between two independent groups were performed using an unpaired two-tailed Student’s *t*-test. Statistical significance was defined as *p* < 0.05.

## 5. Conclusions

The present study demonstrates that substantial minimization of UCOE sequences can be achieved while preserving their ability to support long-term transgene expression in human induced pluripotent stem cells and during neuronal differentiation. By directly comparing three UCOE constructs of different lengths under identical experimental conditions, we showed that the newly developed 0.5 kb UCOE maintained stable eGFP expression over extended culture periods and throughout neuronal lineage commitment, exhibiting performance comparable to that of the larger 1.2 kb and 1.7 kb UCOE fragments.

Importantly, stable vector copy numbers observed throughout the study indicate that sustained reporter gene expression was associated with resistance to transcriptional silencing rather than differences in vector persistence. These findings are consistent with the possibility that the essential functional activity of UCOEs may be retained within a substantially smaller region than previously recognized, although the molecular basis of this activity remains to be determined.

From a translational perspective, reducing the size of UCOE regulatory elements provides additional cloning capacity within lentiviral vectors, thereby facilitating the incorporation of larger therapeutic genes, complex regulatory cassettes, and emerging genome engineering technologies. Thus, the compact size of the 0.5 kb UCOE may provide a practical vector-design advantage by reducing the regulatory cassette footprint. Importantly, however, this structural advantage should not be interpreted as evidence of improved genomic safety. The present study did not directly assess integration-site distribution, insertional effects, genotoxicity, or cellular transformation. The ability of the minimized UCOE to maintain transgene expression during neuronal differentiation further highlights its potential utility for stem cell engineering, regenerative medicine, and gene therapy strategies targeting neurological disorders.

Although additional mechanistic studies are required to define the precise epigenetic basis of its activity and to validate its performance in vivo and across different cellular systems, the present work provides experimental evidence supporting the development of compact chromatin-opening elements. Importantly, the reduced size of the 0.5 kb UCOE should be considered a potential vector-design advantage rather than a demons trated safety benefit, as genomic safety, integration-site distribution, insertional effects, genotoxicity, and cellular transformation were not directly assessed in the present study. Future studies will therefore be required to evaluate the long-term genomic safety and in vivo performance of this compact UCOE configuration. Collectively, our findings identify the 0.5 kb UCOE as a candidate compact regulatory element for further evaluation in lentiviral vector engineering, offering a practical strategy to support long-term transgene stability while maximizing vector design flexibility.

## Data Availability

The data that support the findings of this study are available from the corresponding author upon reasonable request. UCOE sequences are not publicly available due to institutional restrictions; inquiries regarding sequence access should be directed to the corresponding author.

## Supplementary Materials

The following supporting information can be downloaded at: https://www.mdpi.com/article/10.3390/ijms27188046/s1.

## Abbreviations

A2UCOE	A2 Ubiquitous Chromatin Opening Element
CHO	Chinese Hamster Ovary
CHO-K1	Chinese Hamster Ovary-K1
CBX3	Chromobox Protein Homolog 3
CMV	Cytomegalovirus
DNA	Deoxyribonucleic Acid
eGFP	Enhanced Green Fluorescent Protein
FACS	Fluorescence-Activated Cell Sorting
HEK293T	Human Embryonic Kidney 293T
HNRPA2B1	Heterogeneous Nuclear Ribonucleoprotein A2/B1
IgG	Immunoglobulin G
MFI	Mean Fluorescence Intensity
PCR	Polymerase Chain Reaction
PEI	Polyethylenimine
qPCR	Quantitative Polymerase Chain Reaction
RNA	Ribonucleic Acid
RT-qPCR	Reverse Transcription Quantitative Polymerase Chain Reaction
SV40	Simian Virus 40
TBP	TATA-Binding Protein
UCOE	Ubiquitous Chromatin Opening Element
VCN	Vector Copy Number
WPRE	Woodchuck Hepatitis Virus Posttranscriptional Regulatory Element
