# Supplementary material for: Minimal CBX3-Derived UCOE Confers Long-Term Resistance to Transgene Silencing in Human iPSCs During Neuronal Differentiation
Source: Int J Mol Sci. 2026 Sep 10;27(18):8046. doi: 10.3390/ijms27188046 (PMC13606736; doi:10.3390/ijms27188046)
Supplement: Supplementary file 1 [file ijms-27-08046-s001.zip › ijms-4488998-supplementary.pdf]

Supplementary Figure S1

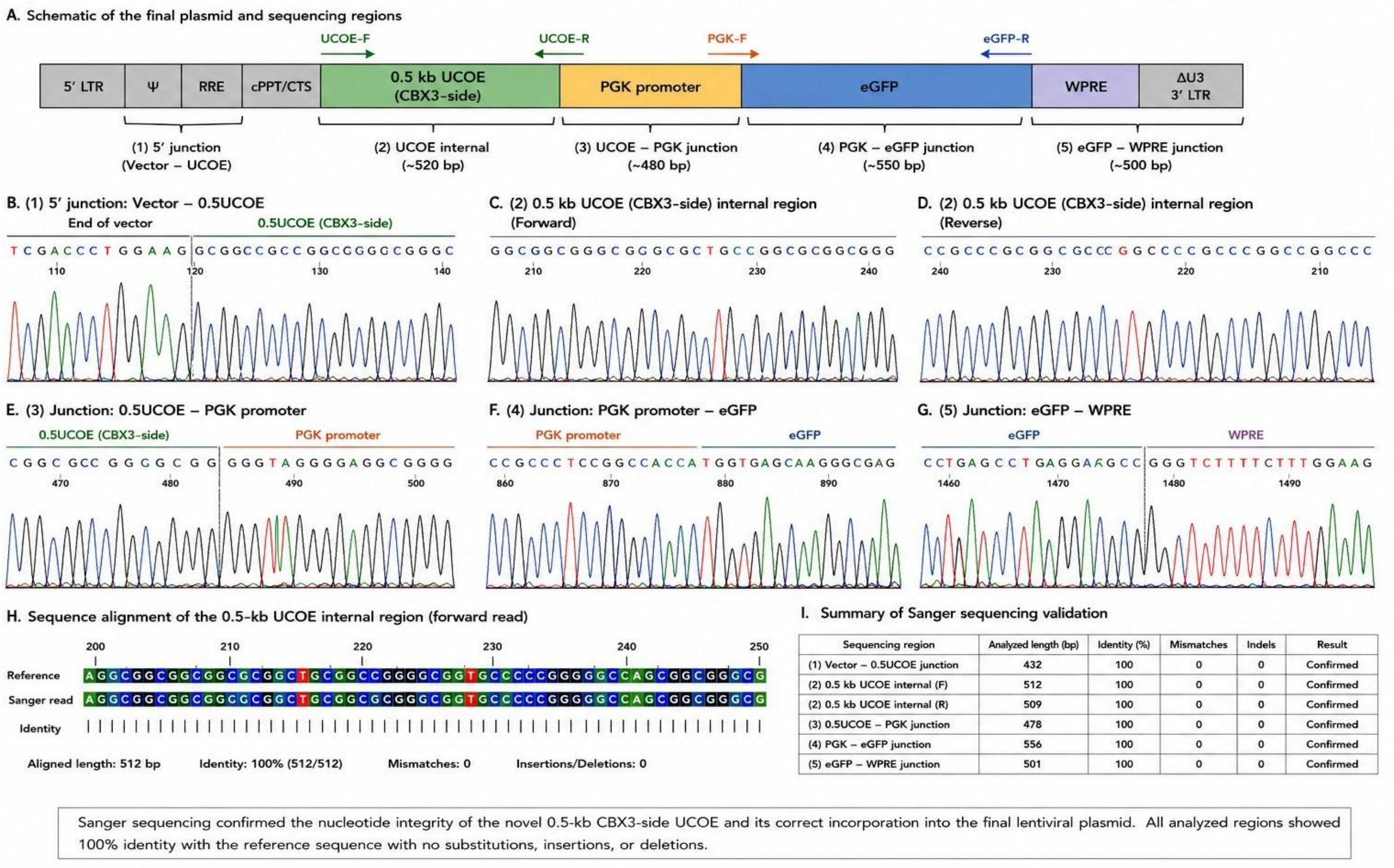

Figure S1: Nucleotide-level validation of the novel 0.5-kb CBX3-side UCOE and final recombinant lentiviral plasmid by Sanger sequencing.

A. Schematic of the final plasmid and sequencing regions B. (1) 5' junction: Vector - 0.5UCOE C. (2) 0.5 kb UCOE (CBX3-side) internal region (Forward) D. (2) 0.5 kb UCOE (CBX3-side) internal region (Reverse) E. (3) Junction: 0.5UCOE - PGK promoter F. (4) Junction: PGK promoter - eGFP G. (5) Junction: eGFP - WPRE H. Sequence alignment of the 0.5-kb UCOE internal region (forward read) I. Summary of Sanger sequencing validation

Sanger sequencing confirmed the nucleotide integrity of the novel 0.5-kb CBX3-side UCOE and its correct incorporation into the final lentiviral plasmid. All analyzed regions showed 100% identity with the reference sequence with no substitutions, insertions, or deletions.

Supplementary Figure S2

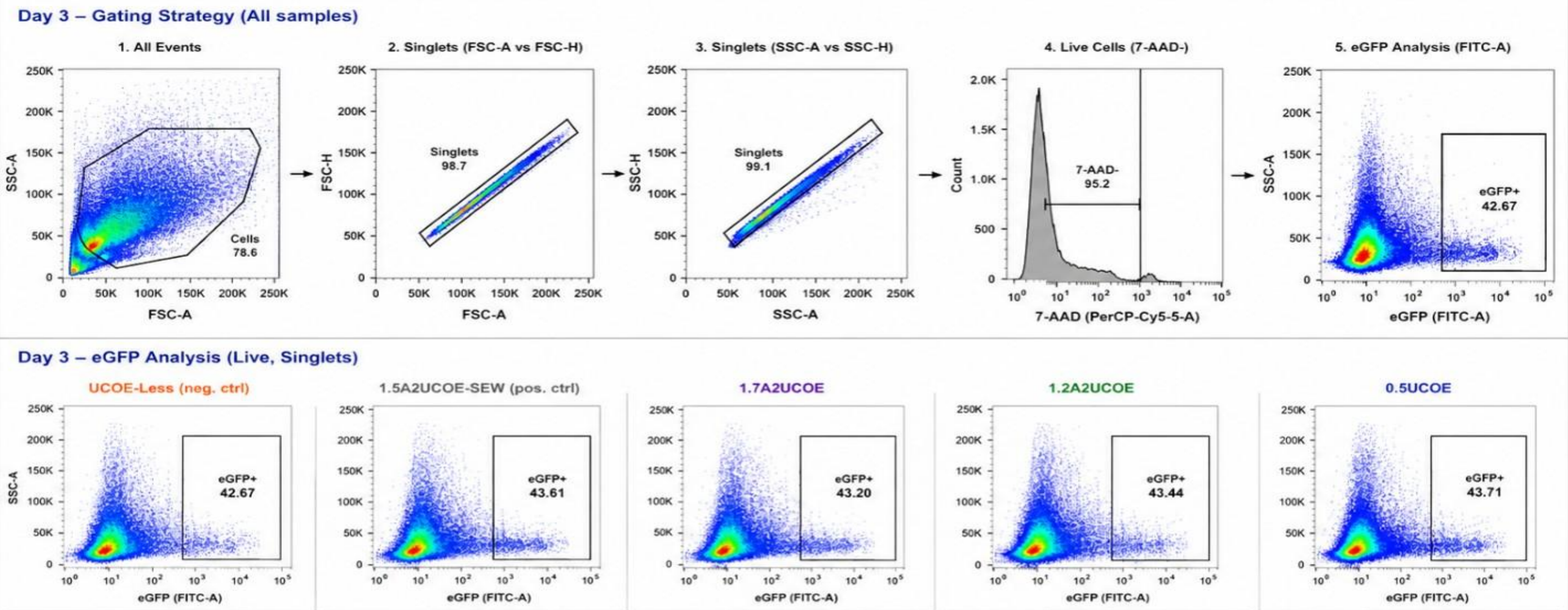

UCOE-Less (neg. ctrl)

Population	#Events	% of Parent	% of Total
All Events	100,000	100.0	100.0
Cells	78,551	78.6	78.6
Singlets (FSC)	77,515	98.7	77.5
Singlets (SSC)	76,878	99.2	76.9
Live (7-AAD-)	73,157	95.2	73.2
eGFP+	31,218	42.67	31.2
eGFP-	41,939	57.33	41.9

1.5A2UCOE-SEW (pos. ctrl)

Population	#Events	% of Parent	% of Total
All Events	100,000	100.0	100.0
Cells	77,642	77.6	77.6
Singlets (FSC)	76,872	99.0	76.9
Singlets (SSC)	76,284	99.2	76.3
Live (7-AAD-)	72,531	95.1	72.5
eGFP+	31,623	43.61	31.6
eGFP-	40,908	56.39	40.9

1.7A2UCOE

Population	#Events	% of Parent	% of Total
All Events	100,000	100.0	100.0
Cells	78,263	78.3	78.3
Singlets (FSC)	77,501	99.0	77.5
Singlets (SSC)	76,864	99.2	76.9
Live (7-AAD-)	73,007	95.0	73.0
eGFP+	31,546	43.20	31.5
eGFP-	41,461	56.80	41.5

1.2A2UCOE

Population	#Events	% of Parent	% of Total
All Events	100,000	100.0	100.0
Cells	78,106	78.1	78.1
Singlets (FSC)	77,265	98.9	77.3
Singlets (SSC)	76,606	99.1	76.6
Live (7-AAD-)	72,742	94.9	72.7
eGFP+	31,579	43.44	31.6
eGFP-	41,163	56.59	41.2

0.5UCOE

Population	#Events	% of Parent	% of Total
All Events	100,000	100.0	100.0
Cells	78,417	78.4	78.4
Singlets (FSC)	77,628	99.0	77.6
Singlets (SSC)	76,955	99.1	77.0
Live (7-AAD-)	73,221	95.1	73.2
eGFP+	31,969	43.71	32.0
eGFP-	41,252	56.29	41.3

Gating: Cells (FSC-A vs SSC-A) → Singlets (FSC-H vs FSC-A) → Singlets (SSC-H vs SSC-A) → Live (7-AAD negative) → eGFP analysis (FITC-A).
Data are from one representative experiment. Untransduced cells were used to set the eGFP-positive gate.

Figure S2: Day 3 Flow Cytometry Gating Strategy and eGFP Analysis

Supplementary Figure S3

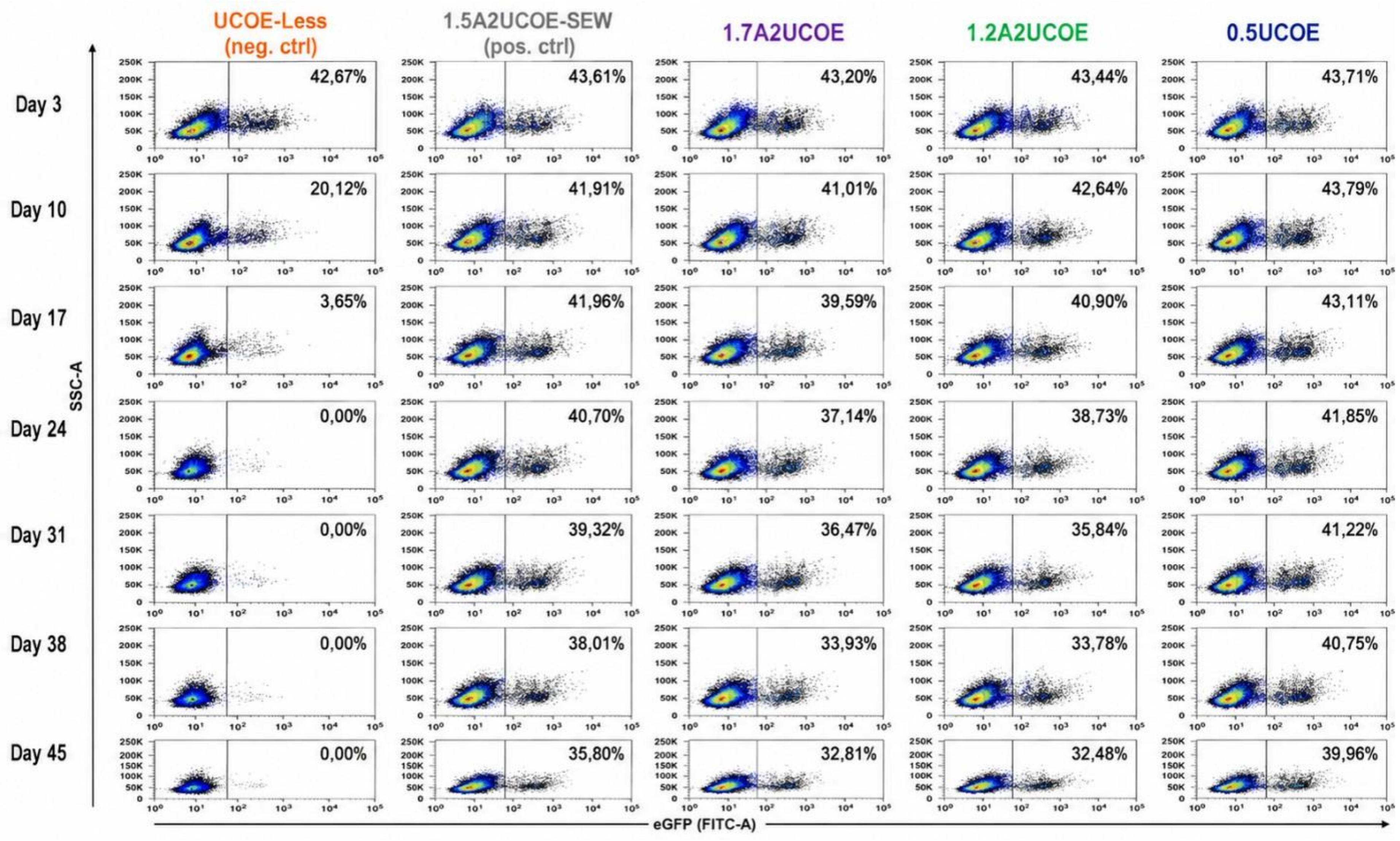

Figure S3: Flow cytometric analysis of long-term eGFP expression in undifferentiated human iPSCs during 45 days of culture.

Supplementary Figure S4

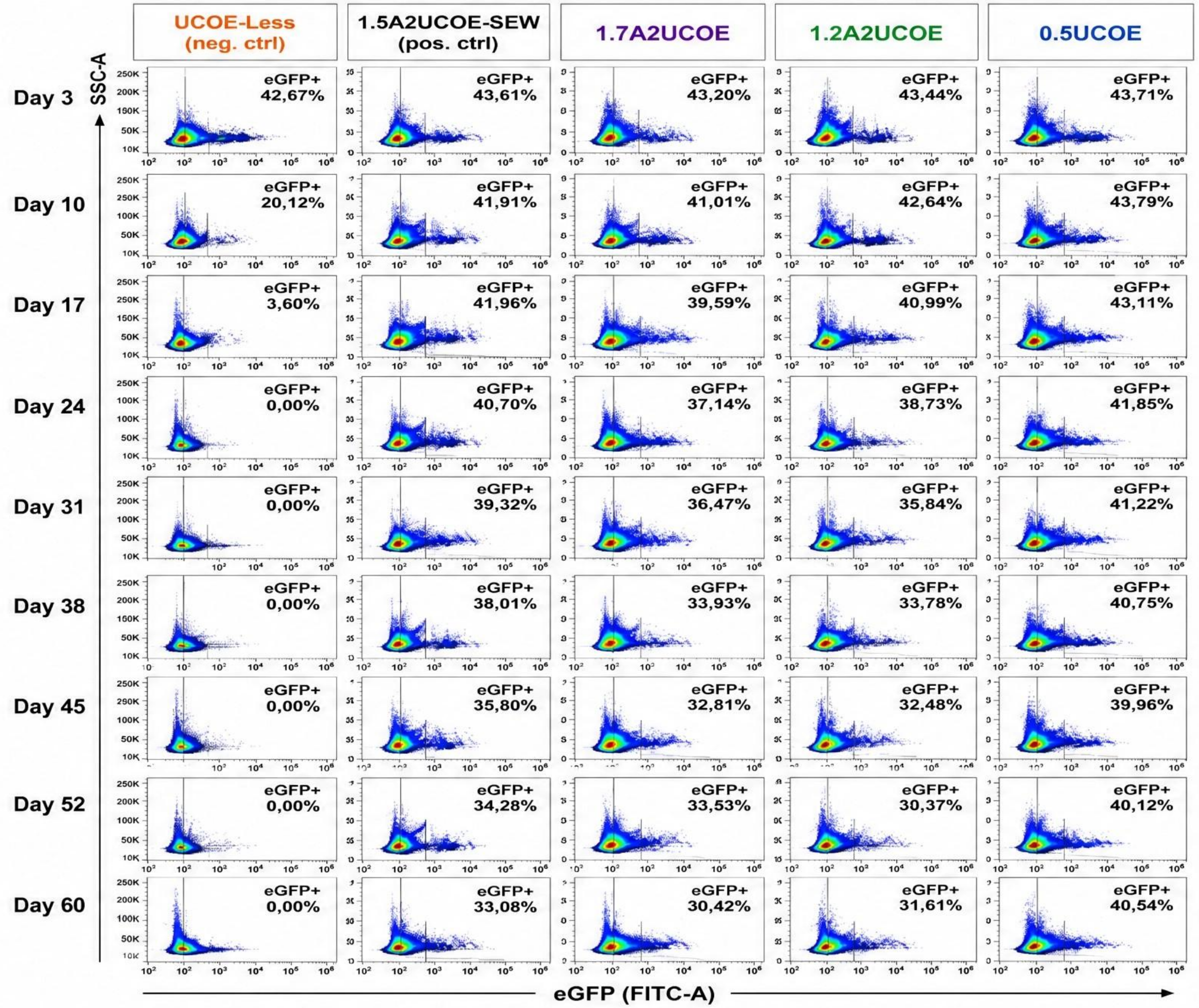

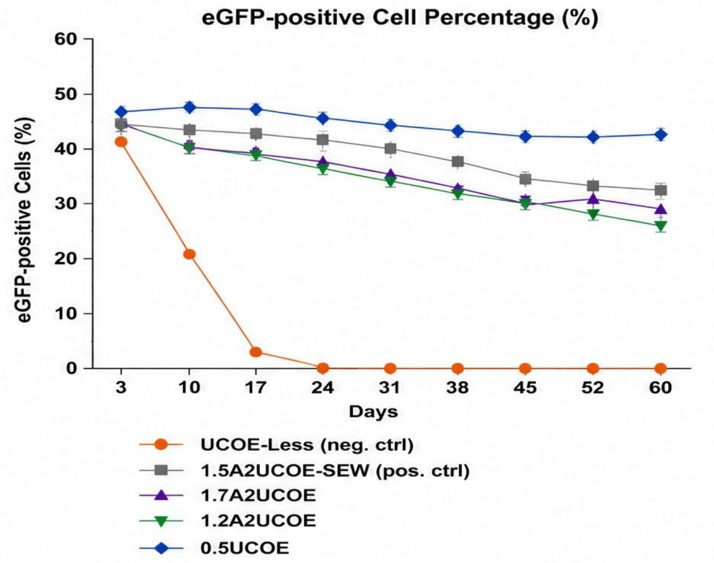

Day	eGFP-positive Cells (%)				
	UCOE-Less (neg. ctrl)	1.5A2UCOE-SEW (pos. ctrl)	1.7A2UCOE	1.2A2UCOE	0.5UCOE
3	42,67	43,61	43,20	43,44	43,71
10	20,12	41,91	41,01	42,64	43,79
17	3,60	41,96	39,59	40,99	43,11
24	0	40,70	37,14	38,73	41,85
31	0	39,32	36,47	35,84	41,22
38	0	38,01	33,93	33,78	40,75
45	0	35,80	32,81	32,48	39,96
52	0	34,28	33,53	30,37	40,12
60	0	33,08	30,42	31,61	40,54

Plots show SSC-A vs eGFP (FITC-A) after exclusion of debris and doublets.
Vertical line indicates the eGFP-positive gate, defined using the untransduced (negative) control.
Data are mean ± SEM (n = 3 independent experiments).

Figure S4: Longitudinal quantification of eGFP-positive human iPSCs during 60 days of culture.

Supplementary Figure S5

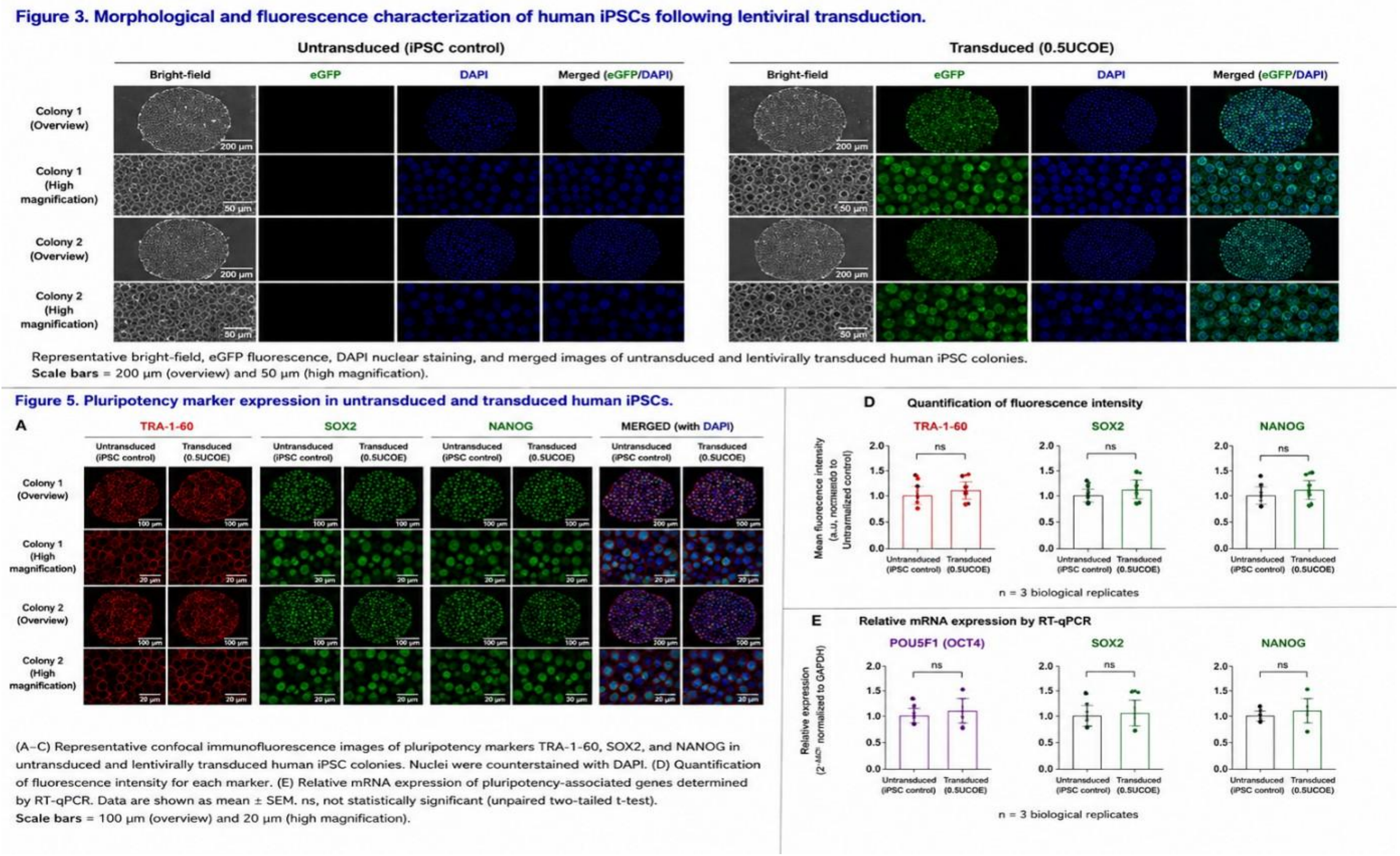

Figure S5: Pluripotency-associated marker expression in untransduced and lentivirally transduced human iPSCs
